# Improving strategic planning for nature: Panacea or pandora’s box for the built and natural environment?

**DOI:** 10.1007/s13280-024-01995-9

**Published:** 2024-03-15

**Authors:** Alister Scott, Matthew Kirby

**Affiliations:** Department of Geography and Environmental Sciences, Northumbria University, Room, B305 Ellison Building, Newcastle upon Tyne, NE1 8ST UK

**Keywords:** Governance, Landscape-scale, Mainstreaming nature, Politics: partnerships, Strategic planning

## Abstract

This paper assesses how strategic planning for nature can be improved for England’s built and natural environment using mainstreaming and landscape-scale concepts. Whilst both concepts feature in academic literature, there has been limited attention on their role as catalytic agents for strategic planning. Addressing this gap, evidence is used from two stakeholder workshops involving 62 senior policy experts managing a range of operational and hypothetical strategic spatial planning challenges. The results reveal a significantly weakened strategic planning arena characterised by policy disintegration, short termism and uncertainty. Key findings highlight the fallacy of pursuing strategic planning for nature in isolation from wider policy integration fusing environmental, economic and social components from the outset. Current barriers to progress include institutional inertia, technocratic vocabularies and neoliberalist priorities exacerbated by a weak underlying theory. Conversely opportunities for mainstreaming processes may help knowledge generation and exchange within transdisciplinary partnerships, whilst landscape scale thinking can improve understanding of issues using natures inherent geometry transforming processes and outcomes. The paper recommends the adoption of strategic planning pathways using mainstreaming and landscape-scale approaches working in tandem. Whilst focused on the English context, our findings are transferable to other planning systems in the Global North, especially those championing neoliberal market led policies.

**Supplementary Information:**

The online version contains supplementary material available at 10.1007/s13280-024-01995-9.

## Introduction

Strategic planning is a heavily used concept in planning research and policy; one which is often presented as a panacea to address societal challenges but without well-developed theory (Albrechts 2016) or identification of the mechanisms necessary for delivery (Trygg and Wenander 2022). Albrechts (2015:511) provides a useful starting point for unpacking strategic planning processes and outcomes as:

*“a socio-spatial process through which a range of participants in diverse institutional relations and positions come together to design plan-making processes and develop contents and strategies for the management of spatial change; an opportunity for constructing new ideas and processes that can carry them forward; collective efforts to reimagine a city, urban region, or region and to translate the outcome into priorities for area investment, conservation measures, strategic infrastructure investments, and principles of land-use regulation”.*

This definition implies logic and order, neglecting the conceptual and practical tensions and inherent messiness within strategic planning inhibiting its potential. First, there is the goal for strategic planning to create certainty, but at a time when planning is characterised by uncertainty and rapid change (Hillier 2016). Furthermore, most planning tools have been designed to deal with situations of stability rather than change (Schön 1971). Second, there is political tension between the desire to create pro-active long-term visions versus the need to react to short-term priorities leading to conflicting temporal priorities (Haughton et al. 2013). Finally, there is a conceptual tension around the need for more radical and participatory planning epistemologies challenging established power structures as opposed to incremental approaches working within existing political and economic structures (Allmendinger and Haughton 2010; Trygg and Wenander 2022). Such tensions are evident in many European planning systems through the widespread adoption of neoliberal approaches which increasingly undermine strategic planning rationales in favour of short-term gains (Loh and Sami 2013; Tait and Hansen 2013; Olesen 2014).

This aim of this paper is to assess how these tensions might be addressed in strategic planning processes using a natural environment lens focussing on the concepts of mainstreaming and landscape-scale thinking, collectively as change catalysts. First, we unpack the core ingredients and associated tensions of strategic planning building on Albrechts (2015) definition set within a wider literature review of strategic planning theory and practice. Second, we assess the contribution of mainstreaming and landscape-scale approaches as potential catalysts to unlock strategic planning potential for nature conservation. Third, drawing on the previous outcomes, we present primary research involving two workshops with professional insights on how strategic planning processes for nature conservation might be improved in England. Finally, there is a critical discussion of how design and delivery of strategic planning for nature might proceed, moving from concept to challenges/opportunities through to delivery.

## Core ingredients and tensions in strategic planning

From both theoretical and practical perspectives, strategic planning is elusive due to its multidimensional nature and its socio-political and institutional complexity which, in turn, is influenced by power configurations, making it highly context and place-dependent (Hersperger et al 2019). Within England strategic planning initially lay at the heart the post-war statutory planning framework evolving into structure plans in 1968 and voluntary regional strategies in the 1970s. But as ties with Europe grew stronger, planning became increasingly influenced by the European Spatial Development Perspective. Thus in 2004, Regional Spatial Strategies were enacted delivered by regional assemblies with statutory planning powers. However in 2010, the Conservative coalition government abolished these strategies in favour of market-led neoliberal traditions. Some re-emergence of strategic planning has evolved though devolution arrangements enabling some combined authorities to secure planning powers but this is happening on an ad hoc rather than ap planned manner (Boddy and Hickman 2013; Lingua 2018) Currently there are increasing calls for strategic planning to be more widely adopted but seemingly still resisted. This inherently political process inevitably generates tensions, paradoxes and dilemmas, which provide strong motivation for this paper.

Building from Albrecht’s (2015) definition cited earlier, strategic planning involves multiple actors coming together from different policy domains to manage change. The nature of that change (transformative or incremental) critically depends on governance and leadership credentials (Lockwood et al. 2010; Van Dijk 2021). Here there is a tension and complex relationship between long-term determination and short-term adaptiveness in strategy formulation (Van Dijk 2021). Shared visions require effective leadership to help functional areas come together to tackle problems in partnership, enabling stakeholders to go outside established comfort zones (Scott et al. 2018). Forester (2010) highlights an opportunity space where negotiation, dialogue and contestation intersect with diverse interests, needs and perceptions and where core values and power relationships shape and/or distort increasingly messy and contested outcomes. However, Mcphearson et al. (2021) argue for more radical transformative thinking and agendas than shared visions currently enable. In pursuit of this the focus has shifted from top-down, expert-led models and approaches to “managed” spaces where top-down and bottom-up approaches converge championing co-design and coproduction (Reed et al 2018). Key to this is more inclusive and interdisciplinary partnership models engaging multiple public(s) and crucially going beyond the ‘usual suspects’ (Cowling et al. 2008). However, this by default challenges decision-makers to allow participants to have a stake in the process and outcomes which may threaten existing power structures (Beunen et al. 2013

Thus, power relations and knowledge(s) become important variables in strategic planning in terms of what evidence is collected, legitimatised and accepted; and by who and through what processes and outcomes (Mckenzie et al 2014)? Eränta and Mladenović (2021) argue that there is insufficient understanding of the dynamics of such knowledge processes. Notably, spatial evidence bases used to aid decision-making often prioritise instrumental rationality over other forms of evidence production (Sheppard 2005), with a need to better integrate and represent local knowledge(s) (Sui et al. 2013). Dempwolf and Lyles (2012) have identified a lack of empirical knowledge of how individuals in planning processes are embedded in the dynamic networks for addressing the complex societal issues we now face. Furthermore, Savini and Raco (2019) highlight that knowledge is increasingly becoming institutionalised, technocentric and expert led. This ‘political anaesthetic’ then constrains possibilities for political conflict and the generation of more radical alternative forms of intervention thus perpetuating the status quo hindering the policy and scalar integration that strategic planning seeks (Olesen 2014).

Policy integration is crucial to strategic planning processes involving multiple agencies coordinating planning across horizontal (sectors), vertical (spatial) and temporal (time) scales (Tewdwr Jones et al., 2010). The scalar dimension shifts attention towards more challenge-led rather than agency-led agendas However, Stead and Meijers (2009) highlight the importance of political, institutional, economic and instrumental factors as shaped through the interpretations and behaviours of key actors. This brings added complexity to the ensuing narratives and power relations between involved actors which influence decision-making processes. Furthermore, the requirement for citizen engagement (Cavaco et al; 2023) poses new governance and accountability challenges for policy integration. Indeed, Mommaas and Janssen (2008) caution against viewing integration as a panacea given risks of compromise and lowest common denominator solutions.

The notion of certainty is also key to strategic planning outcomes, yet uncertainty characterises not only all of the elements within the interconnected web of dynamic perspectives, visions and plans that is constitutive of strategic planning but also the external context, which can develop in unpredictable ways (Hillier 2016).

Regulation is often seen as a magic bullet for strategic planning (UKNEAFO 2014). Yet, it needs to be designed within inclusive participatory processes, together with necessary guidance and resources for delivery, monitoring and enforcement. Regulation also works best when used in tandem with other incentive and participatory tools as bundles (Scott et al. 2014a). But any lack of resources for its delivery and enforcement can readily lead to failure in practice or regulatory capture (Niederburger and Kimble 2011). Furthermore, regulation risks diluting existing standards to what might only be acceptable. Additionally, if regulation is not fully “accepted,” then it may be simply viewed as a hurdle to overcome resulting in tokenism or avoidance strategies.

Regulation is embedded within governance frameworks which design and deliver strategic planning. Here, there is an important distinction between voluntary and statutory strategic planning approaches. Voluntary approaches can be successful in creating innovative visions and broad participation, but they can fail at the implementation phase due to a lack of statutory power and legitimacy (Allred and Chakraborty 2015; Mäntysalo et al. 2015). Conversely, statutory strategic plans can be effective through consistent approaches with appropriate local flexibility (Schmid et al. 2021). A formal regional planning tier can further support strategic planning through dedicated resources and political consensus (Kline et al. 2014). Equally however, non-transparent regulatory and strategic control can erode trust in statutory approaches to strategic planning, which has been attributed to the decline of regional governance in some European systems including the UK (Tait and Hansen 2013). Here the deeply embedded tension between control and laissez-faire rears its head with a need to strike a balance between these two polarities (Van Dijk 2021).

Finally, strategic planning should lead to positive outcomes that identify new opportunities to lever funding and investment. The explicit linking of policy with funding mechanisms and the pursuit of blended finance from the outset are important but often overlooked. Otherwise, this creates a significant policy–delivery gap. Indeed, delivery in its widest context is key to a successful strategic planning process; its capacity to produce action frameworks and ability to mobilise people to action (Albrechts 2015). Crucially, these action plans need to identify who does what, when and how (Dyrberg 1997). It is also important that those involved in delivery are also involved in the strategic planning process in order to minimise the risk of overambition and under delivery (Scott et al. 2013).

## Strategic planning for nature conservation: a conceptual framework

‘Nature across most of the globe has now been significantly altered by multiple human drivers, with the great majority of indicators of ecosystems and biodiversity showing rapid decline. Seventy-five per cent of the land surface is significantly altered, 66 per cent of the ocean area is experiencing increasing cumulative impacts, and over 85 per cent of wetlands (area) has been lost.’ (IPBES 2019 A4 XV).

The quote above shows that despite improved evidence, concepts, tools and strategies, the decline in species and ecosystems globally and nationally in England is ongoing, signifying policy and delivery failure(s). Environmental discourses utilise multiple conceptualisations of nature often coexisting and struggling to influence change on the ground (Macnaghten and Urry 1998; Mercado et al. 2024).

Traditionally, the approach to environmental protection has been through designation of key species and habitats within a designation hierarchy with dedicated management plans and advisory committees for the most important landscapes, sites and species. However, more recently there has been a shift towards more holistic approaches using a social–ecological systems perspective involving the re-conceptualisation of the natural environment as an asset leading to financial and non-financial valuations of nature through national ecosystem service assessments (MEA 2005) and the development of green infrastructure networks, nature-based solutions and wider landscape-scale thinking (Ahern and Cole 2012; Mell and Scott 2023; Mercado et al. 2024). These changing foci catalyse strategic planning responses that cross traditional administrative boundaries adding complexity as new governance layers such as ecological networks and water catchments with greater responsibilities for non-governmental actors. However, operating at these larger and unfamiliar spatial scales poses challenges for wider public engagement and support (Beunen et al. 2013).

From this brief overview, we now focus on mainstreaming and landscape-scale concepts that we believe can add value to strategic planning for nature conservation. Currently, these concepts have not been used explicitly in strategic planning theory but arguably they serve as change catalysts to help reshape strategic planning processes both in their own right but also collectively.

### Mainstreaming nature in strategic planning

**Fig. 1:**
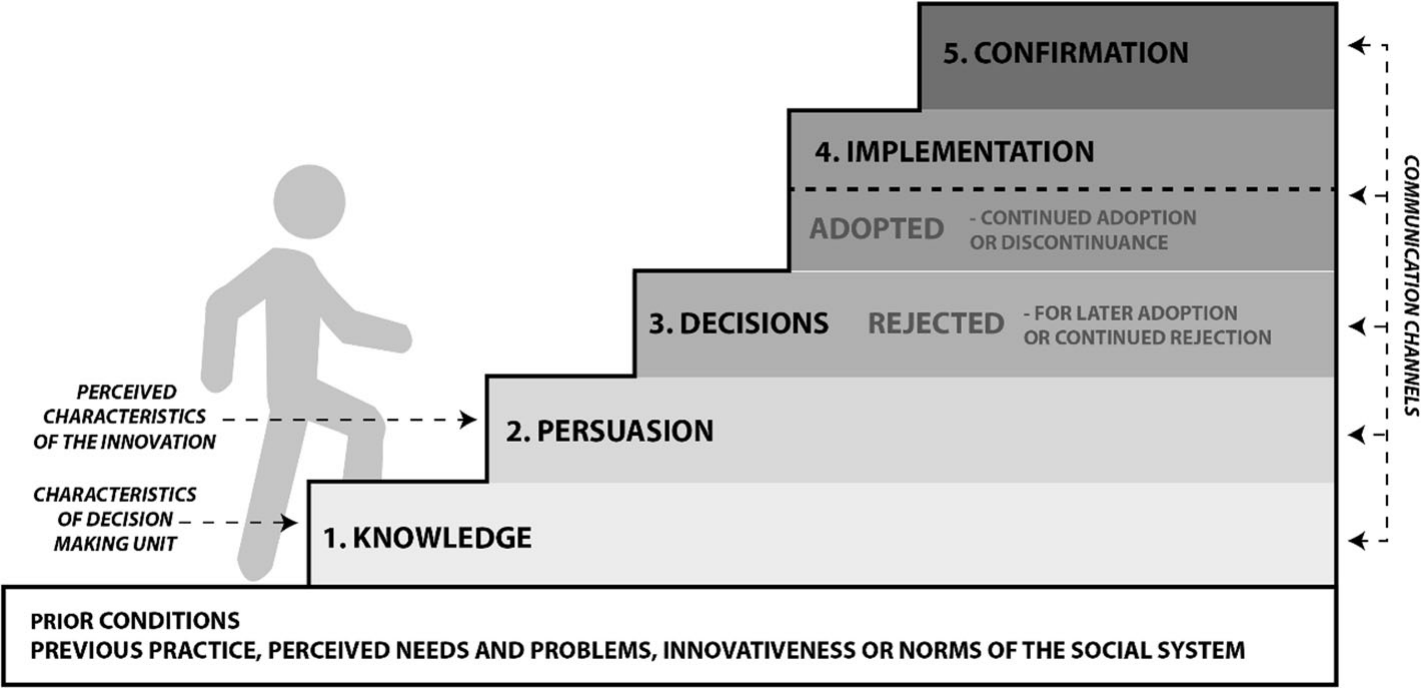
The mainstreaming process: Redrawn from Scott (2019:424)

Mainstreaming nature involves taking key environmental concepts such as natural capital, ecosystem services and nature-based solutions and then translating and/or adapting them so that they become normalised within other policy domains such as housing, economy and transport, where they are not (yet) sufficiently understood and/or accepted by those audiences (Scott et al 2022). This requires overcoming resistance or challenge within those policy sectors who may indeed have their own priorities associated with economic growth or viability (Cowling et al. 2008; Benson et al. 2014). Mainstreaming processes necessarily involve ‘messy’ and reversible pathways of diffusion from initial ideas through persuasion to testing and adoption and or rejection (Fig. 1). Mainstreaming pathways can be shallow and/or deep. Shallow interventions, such as taxes or incentives, are relatively easy to employ, though they only secure minor system changes, which are vulnerable, whereas deeper interventions involve more upfront investment and are more value-based given their emphasis on collaborative working and co-production, which may result in more transformative behaviour change (Scott et al. 2022).

Environmental mainstreaming in strategic planning has had limited success (Scott et al. 2022). Concerted efforts championing neoliberal traditions of nature as assets for development using ecosystem services and natural capital through nationwide and global assessments have led to incremental policy change (NEAFO 2014; Maes et al. 2020). Benson et al. (2014) argue that mainstreaming nature across national policies, sector plans and budgeting processes may gain more success if led by, or collaborated with, more influential ministries of planning and/or finance. In England, the influential DasGupta (2021) Review on Biodiversity commissioned through the UK Treasury provides a useful albeit controversial example of this approach to mainstreaming. However, Spash and Hache (2022) argue that the current predilection for valuing and pricing nature to optimise resource management serves only to prioritise wealth accumulation and maintain business-as-usual, rather than improve outcomes for nature. Indeed, Mercado et al. (2024:80) see anthropogenic narratives “imbued with significant ontological and epistemological assumptions which may constrain rather than enable wider processes of change”. Such criticism links in with other commentators who use a political ecology lens to highlight problems with strategic planning practices for nature (Hurley and Walker 2004). Here, Mercado et al. (2024) argue for a reconceptualisation of “nature with people- not for people” as a reaction to neoliberal narratives of nature.

Key to mainstreaming endeavours is getting past the persuasion stage (Fig. 1). Scott et al., (2018) proposed ‘hooks and ‘bridges’ mechanisms to bring specialised and general audiences together respectively to help garner initial traction. For example, the use of climate emergency, health inequalities, place-based approaches and well-being, are seen to have strategic planning currency as bridges, uniting disparate audiences. Here, the efficacy of communication channels and securing the active support of non- environmental gatekeepers become crucial in making progress (Jordan and Russel 2014). Current governance frameworks have a key role here bringing into focus the dynamics of power relationships and conflict management. Russel et al. (2018) identify how these operate across different levels from individual agency to societal values, stressing the need to study the interactions between levels as much as the levels themselves. However, institutional and gatekeeper inertia may provide significant barriers to desired policy and behaviour change (Kingston and Caballero 2009).

### Landscape-scale and strategic planning for nature conservation.

**Fig. 2:**
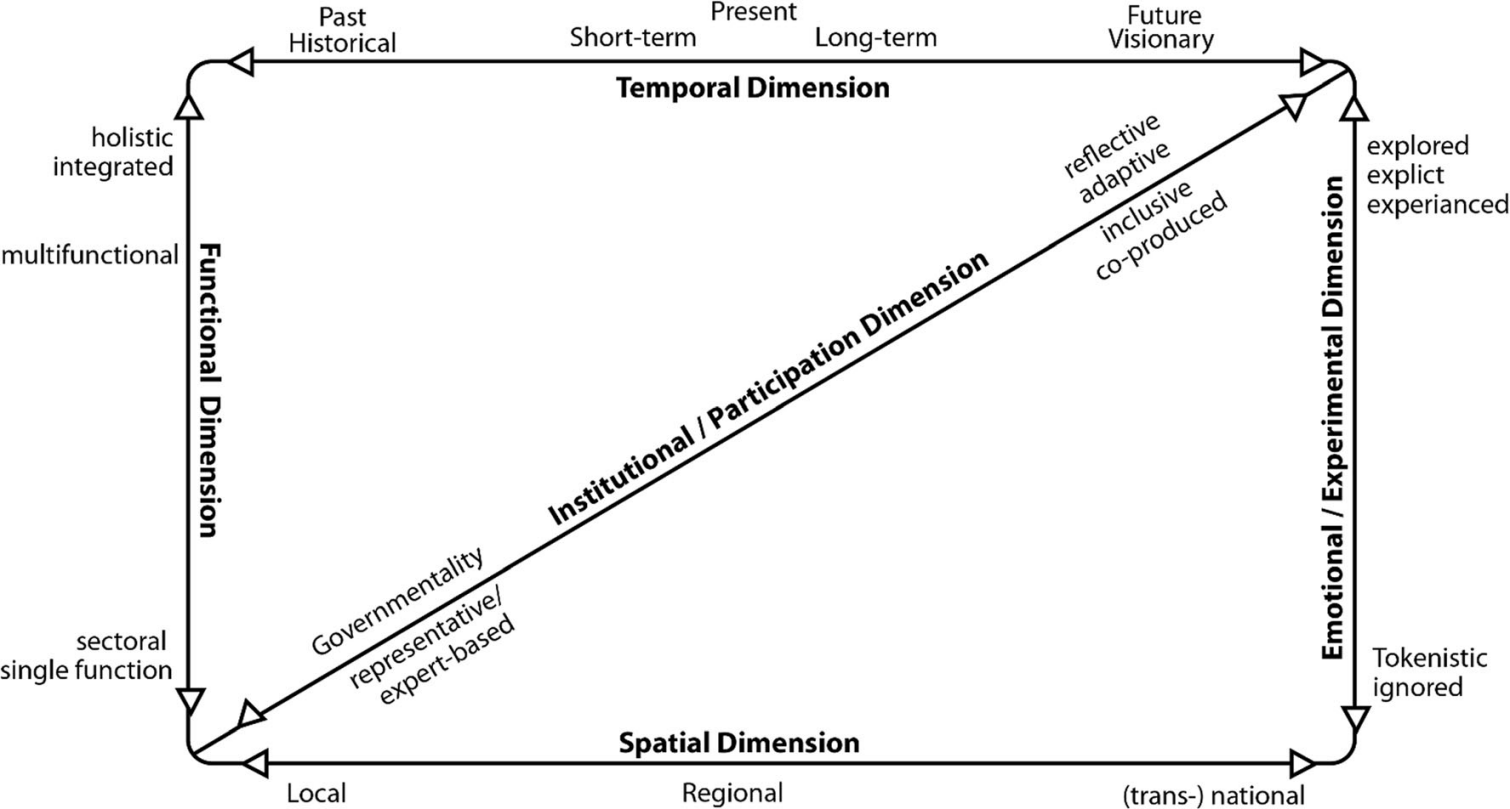
The dimensions of the landscape scale. Adapted from Carter et al. (in press)

Henson et al. (2009: 508) describe the goal of landscape-scale considerations as “*to halt or reverse the process of landscape fragmentation” and “….. to conserve an area large enough to sustain a majority of conservation targets but that is a manageable size for intervention strategies to be applied effectively”.* However, few studies have scrutinised what landscape scale actually means and its role in strategic planning, policy and decision-making leaving it open to vagueness and misrepresentation (Selman 2006; Sayer et al. 2013). Since the 1990s, conservation policy and actions have evolved from a designated site approach to give greater priority to landscape-scale processes (Opdam and Wascher 2004), reflecting drivers of climate change and increased habitat fragmentation. In England, a landmark report ‘‘Making Space for Nature’’ (Lawton et al. 2010) summarised key scientific literature to increase the effectiveness of protected area networks in fragmented landscapes via an effective catchphrase: ‘‘more, bigger, better and joined-up’’. Carter et al., (in press) following a review of current landscape-scale literature have identified five core dimensions which collectively offer significant additionality (Fig. 2).

The spatial dimension remains the dominant focus of landscape-scale research and practice. Authors emphasise the notion of nested scales to describe various landscape functions and processes and how awareness and scrutiny within and between the different scales inform more holistic solutions (Wyborn and Bixler 2013).

The functional dimension champions multifunctionality of landscape-scale plans, ranging from biodiversity conservation to catchment management to ecological networks. Set within this is the challenge of identifying and assessing associated trade-offs which are often neglected in idealistic pursuit of multifunctional goals (Hamback et al., 2023). Many of these initiatives are embedded in a natural resource management context and feature an explicit goal to move from the narrowly-drawn territorial (administrative and jurisdictional) boundaries of land-use planning to adapt to nature’s ‘inherent geometry’ (Bailey 2007).

The temporal dimension shifts focus away from short-term timeframe such as election cycles towards longer-term perspectives to tackle issues such as cumulative impacts and lag effects (Scott et al. 2014b).

The institutional dimension centres around governance and the importance of partnerships as the principal delivery vehicles at the landscape scale (Carter et al., in press). These are heavily prescribed with regulatory aspects dominating, with environmental goals, local knowledge and participatory processes subservient (Beunen et al. 2013). Proponents of the landscape-scale champion the fusing of natural resource management with more collaborative approaches (Berkes 2004) such as community-based natural resource management (CBNRM) (Pailler et al. 2015). The active involvement of citizens and/or communities in this institutional context can deliver multiple benefits including greater autonomy, equality, social capital and democratic values (Mansbridge 1997).

Finally, the emotional dimension captures personal attachments to place, shaping cultural values and social memories, which can generate contested narratives with top-down notions of landscape-scale management and planning (Herbert-Cheshire and Higgins 2004). Consequently, personal, social, expert and lay perspectives all need better embedding in policy and decision-making (Terkenli 2005).

## Materials and methods

**Fig. 3:**
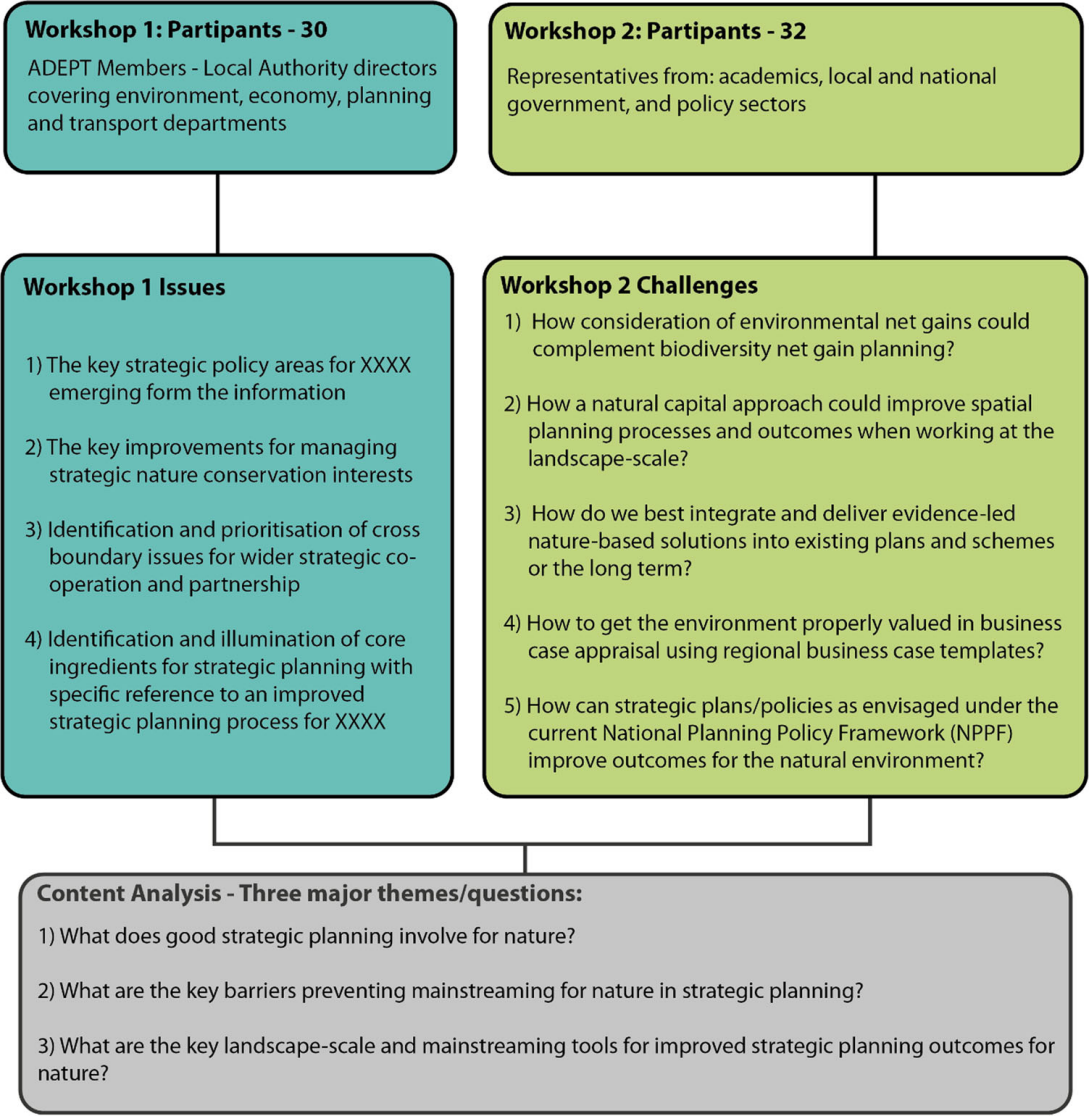
Workshop summary characteristics

This paper uses material from a funded contract to improve strategic planning for nature in England. Two workshops were held in early 2020 with senior professionals in planning policy and practice together with academics across England to better understand current approaches, barriers and opportunities for strategic planning in general and nature conservation in particular. In total, 62 participants were involved in the process (Fig. 3). Ethics approval was obtained from University X Application number 17191 August 2019.

**Fig. 4:**
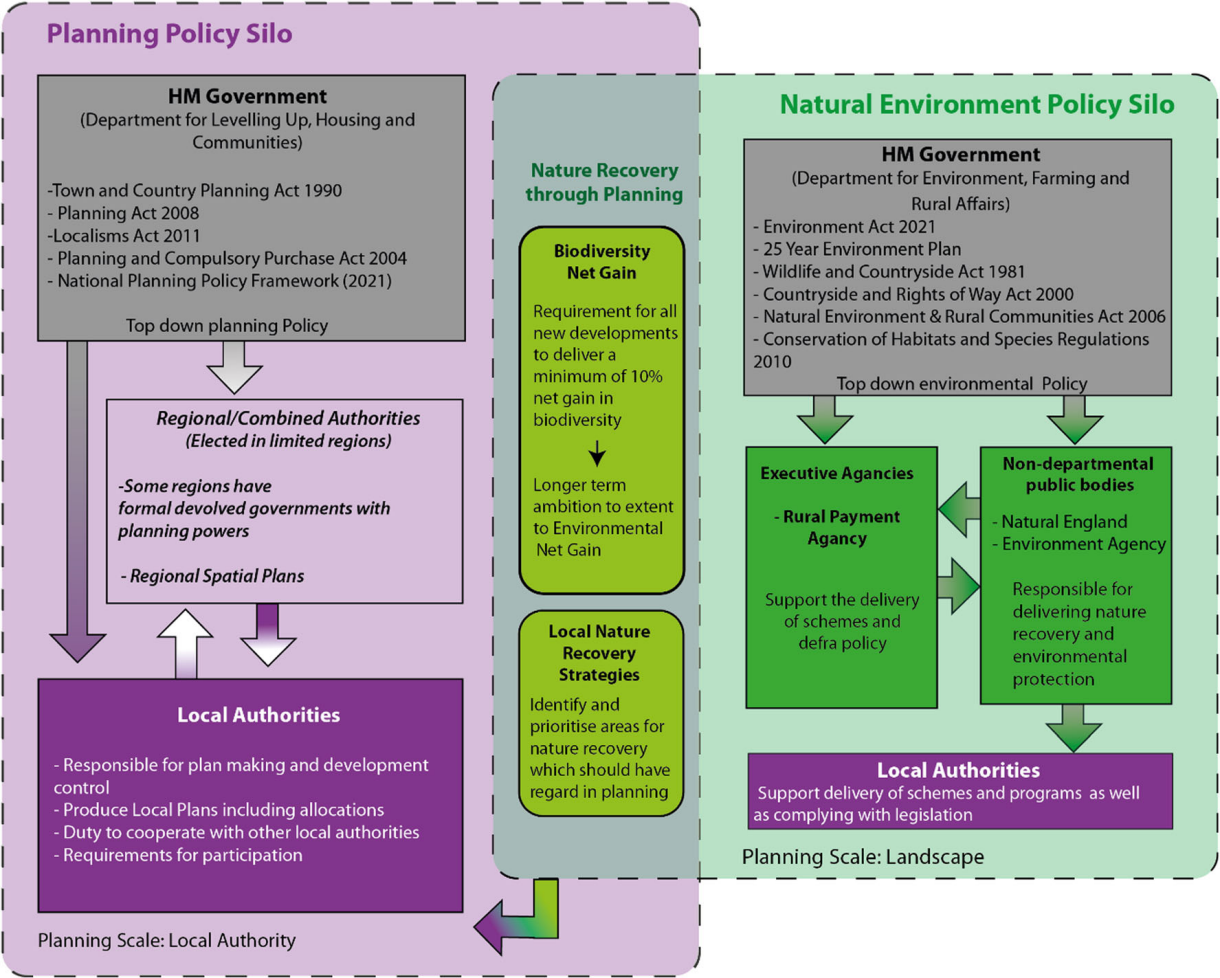
Simplified overview of planning and natural environment policy silos with overlap with recent efforts to deliver nature recovery in the planning process. (*Source*: authors)

Given the English focus of these workshop, it is important to briefly outline the English planning system and the role of nature within it as outlined in Fig. 4. England has a plan-led system where planning policy is delivered through statutory local authority (LA) development plans guided by central government policy (National Planning Policy Framework and National Planning Practice Guidance). Currently, strategic planning is undertaken by LAs through the Duty to Cooperate function which requires cooperation on matters of cross boundary importance under the Localism Act 2012. Additionally, combined authorities where planning powers exist as a result of the devolution agreement can have planning powers (e.g. Greater Manchester Combined Authority) and formal regional planning of London through the Greater London Authority. Whereas strategic planning for nature is formally the responsibility of the Department for Environment, Farming and Rural Affairs, since the workshops there have been notable attempts recently to deliver nature recovery through the planning system in the form of Local Nature Recovery Strategies and requirements for Biodiversity Net Gain (early 2024).

### Workshop 1 Association of Directors of Environment, Economy, Planning and Transport (ADEPT) 3)

This workshop was structured around 5 five tables of six participants working in groups within a 3.5-hour session to collectively identify and discuss four issues (Fig. 3). It took place on 29th January 2020 involving 30 participants from ADEPT. These were all local authority directors covering environment, economy, planning and transport departments. These participants with their integrated and leadership remits were ideally placed as they directly inputted into strategic planning within their local authorities within their leadership roles. The session was structured around a briefing note (Supplementary Evidence A) on a hypothetical region RUFshire within which 28 different challenges/scenarios were set. The adaptation of a hypothetical game for the workshop session created a safe hypothetical space enabling a free-flowing discussion based on the issues in RUFshire  rather than confronting ADEPT member place sensitivities, which might restrict discussion. Recruitment was made through the secretariat of ADEPT sending an invite for the workshop in London. Participants were primarily from the economic development and sustainability working groups.

The discussions were captured on each table by participants themselves via flip charts with the feedback session outputs recorded by the lead facilitator (author). A combined summary report was circulated back to participants for further comment leading to a final report (Supplementary Information B).

### Workshop 2 Improving mainstreaming of nature in strategic planning policy

The second workshop was structured around five different strategic environmental planning challenges (Fig. 3). These challenges were created based on author-led prioritisation of key environmental challenges from UKRI-funded knowledge exchange grant on mainstreaming nature. This workshop was held in London on 28th January 2020 involving 32 participants. Participants were targeted for each challenge including a mix of academic (10), government (4) and policy sectors (18). Representatives were from England with 1 Scottish representative. Each challenge had a lead facilitator and note taker with discussion lasting 3 hours including a feedback and plenary session. Notes were taken with the feedback session and plenary discussion recorded. A summary was sent back to participants for further comments and a final report agreed (Supplementary Evidence C).

Whilst both workshops were held nearly 4 years ago, the issues discussed have a contemporary relevance to current planning debates and the re-emergence of the need for improved strategic planning to address current crises in nature, economy and well-being.

From the recorded observations and written summaries, the two workshop outputs were subjected to a simple contents analysis and then filtered through our mainstreaming and landscape-scale lenses to address three major questions (Fig. 3).

1. What does good strategic planning involve for nature ?
2. What are the key barriers preventing mainstreaming of nature in strategic planning?
3. What are the key landscape-scale and mainstreaming tools for improved strategic planning outcomes for nature.

These challenges are unpacked in the results section below within a narrative referencing workshop 1 or 2 material as justification.^1^ Quoted material reflects specific items recorded on flipcharts and/or written summaries and/or in recorded feedback discussion.

## Results

### What does good strategic planning involve for nature?

Most workshop participants viewed strategic planning for nature as an oxymoron as strategic planning was more about integrating different land uses, sectors and agencies within a given plan or approach rather than addressing any specific sector’s needs separately. Indeed, sectoral silos were seen to “*provide safe spaces for retreat when confronted with more ‘uncomfortable’ holistic dialogues and visions* “ (Workshop 1).

Workshop 1 highlighted the importance of the political dimension in relegating nature to a secondary consideration. Specifically, the English government’s priority for delivering 300, 000 homes per year was seen as *“distorting the strategic planning process*” and *“starting from the wrong position*” (Workshop 1). Thus, strong political champions and leaders were needed that could integrate disparate interests in partnerships such as Greater Manchester (Workshop 2) across political divides from the outset to build long-term resilience given short-term electoral cycles (Workshop 1). Here co-designed visions with supporting outcomes were seen as important tools to unite different professional audiences (Workshop 1 and 2). “B*eing able to articulate clearly your desired outcomes are fundamental but are often neglected”* (Workshop 1). This requires an upfront and deliberative process of engagement across multiple publics to be successful (Workshop 1 and 2). Interestingly, there was unequivocal rejection of top-down or bottom-up approaches in favour of their convergence within more ‘managed’ and safe spaces (Workshop 1).

### What are the key barriers preventing mainstreaming of nature?

There was clear recognition of seven major barriers hindering mainstreaming of nature in strategic planning. First, the explosive growth of a technocratic environmental vocabulary was seen to confuse and alienate multiple stakeholders (Workshop 2). Second, the increasing complexity of governance frameworks made it increasingly difficult for stakeholders to access and navigate. In Workshop 2, the multiple delivery systems of English planning that operated simultaneously over the same geographical space were exposed. The National Planning Policy Framework (NPPF) informed town and country planning responses; Environmental Land Management Schemes (ELMS) informed resource planning (agriculture and forestry) responses; OFWAT and other regulators informed utility planning responses; National Infrastructure Commission informed national infrastructure planning whilst building services informed building regulations. However, these multiple delivery systems rarely joined up; each possessing their own separate governance frameworks (agencies, goals, objectives, functions and enabling legislation) which (dis)integrates planning across the same space (Workshop 2).

Third, within this disconnected landscape, there were no agencies with integrated remits that are charged with managing the bigger strategic picture (Workshop 1 and 2). Some participants argued that local authorities/combined authorities were best placed to do this, but *“currently do not have the necessary resources and capabilities to do so*” (Workshop 2). Ideas were put forward that the UN sustainable development goals perhaps provided an integrated framework for action but these were not currently being prioritised within UK government (Workshop 1).

Fourth, the NPPF (2019 revised) was agreed as being the principal government framework addressing planning policy and delivery, but it limited strategic planning policy and delivery due to its preoccupation with housing need assessments and a general failure of the duty to cooperate (mandatory cooperation on issues of more than local importance) (Workshop 1 and 2). Furthermore, the growth of permitted development^2^ where the conversion of commercial buildings to residential buildings without planning permission conflicted with strategic planning goals for residential development as they do not always have necessary infrastructure and services in place (Workshop 1 and 2).

Fifth, whilst the ambition for nature was recognised in many local authority development plans, this was constrained by relatively weaker policy wording/actions reflecting priorities given towards economic growth (Workshop 1 and 2).

Sixth, the operation of metrics/targets hindered strategic planning. In particular, quantity based environmental metrics such as tree planting which were often used to address the climate change emergency and help biodiversity. “*The numbers involved will be very expensive and seem more about a short term being seen to do something factor than good strategic planning *per se “ (Workshop 2). Furthermore, there was more support voiced for managing our existing woodland stocks better rather than focusing on “dodgy” planting metrics with the concomitant risk that tree planting may not be in the best places.

Finally, there was a key barrier associated with staff capacity and capability on biodiversity matters who could adequately assess, interpret and communicate evidence to external stakeholders, elected members and public in policy formation and public engagement exercises (Workshop 2).

### What are the key landscape-scale and mainstreaming tools for improved strategic planning outcomes for nature?

Effective public engagement and knowledge exchange were seen as key tools to aid landscape scale and mainstreaming processes but only if they happen at the earliest opportunity and involve both the usual and “unusual” suspects. Furthermore, they need to be carefully co-designed and bounded limiting wish lists as well as addressing all legitimate strategic concerns. Workshop 1 questioned the resources and time spent on public consultations with a desire for more purposive and pragmatic responses, although statutory consultation procedures should still be respected. Conversely it was felt that engagement needed to be managed over phases as a deliberative process, set with an evidence-led dialogue better capturing and harnessing both expert and local knowledge(s) across sectors (Workshop 1 and 2).

UK parliament declared a climate emergency in November 2019, and this was seen as a powerful hook for mainstreaming nature. Together with overwhelming evidence of a biodiversity emergency, there was a combined opportunity for government to embed climate and biodiversity as core strategic issues alongside emerging tools of net gain and nature recovery in a revised NPPF alongside housing and economic growth (Workshop 1 and Workshop 2). At the time of writing December 2023, we still await a revised NPPF.

The proposed statutory requirement for Biodiversity Net Gain (Environment Bill 2020) was generally welcomed to help “*level up the playing field* (Workshop 1). However, there were important caveats associated with protections needing to be in place to prevent site damage before baseline net gain assessments were made and concern that development should respect the mitigation hierarchy which was perceived to be commonly bypassed (Workshop 2).

In both workshops, there was concern that all too often research generated new tools rather than assessed whether current tools were fit for purpose and how they might be used for improved nature conservation outcomes (Workshop 1 and 2). Thus, both workshops focused on opportunities for existing tools that were seen as offering best potential for improving strategic planning processes and outcomes for nature.

The first family of tools were impact assessments (Strategic Environmental Assessment. Habitat Regulatory Assessment, Species Regulatory Assessment and Sustainability Assessments). There was agreement that these tools were not being used to their maximum potential for nature. Firstly, they were commonly viewed as hurdles to overcome rather than as tools to improve plan/policy outcomes (Workshop 1 and 2). Secondly, they often became tick-box exercises rather than important parts of a process adding value (Workshop 2). Thirdly, due to cuts in local authority resources, they were consultant-led; consequently separated from other strategic planning work that impact assessments were supposed to inform (Workshop 2). Fourthly, they were often misused to confirm a preferred policy option rather than to help shape the best policy and planning response (Workshop 2). Finally, within impact assessment processes there was limited attention on developing suitable alternatives leading to rejection of plans by the Planning Inspectorate^3^ (Workshop 1).

The second family of tools were associated with betterment. Here Section 106 planning agreements which deal with securing community and environmental benefits tied to a particular development and the Community Infrastructure Levy (CIL) which deals with wider benefits which can be off site and more strategic. For many participants CIL represented a missed strategic opportunity for improved nature conservation outcomes (Workshop 1) as CIL can be used to invest in infrastructure unrelated to a proposed development and pooled to optimise societal benefit whereas Section  106 planning agreements are necessarily tied to a specific development site (Workshop 1). Participants felt that the climate, biodiversity and health emergencies now represented potent policy hooks for CIL investment although not all authorities have a CIL and the status of this levy is uncertain.

Both these tools illuminate a mainstreaming and landscape-scale challenge in that nature conservation is currently a bolt-on process set within a constraint to overcome. This raises the need to change the wider culture in how nature and the tools themselves are viewed, operated and delivered.

## Discussion

**Fig. 5:**
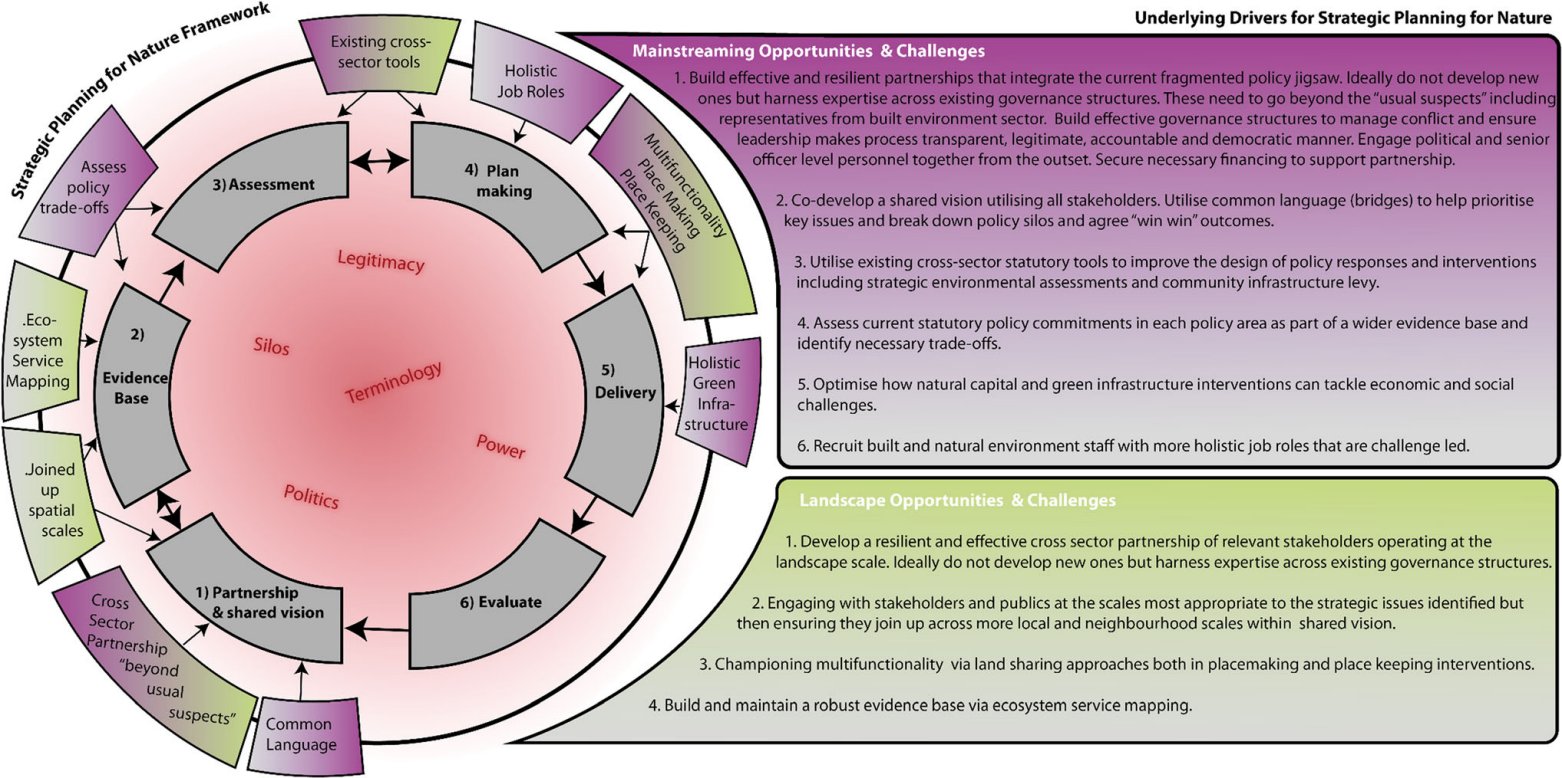
Conceptual framework for strategic planning for nature emphasising the landscape opportunities/barriers and the mainstreaming opportunities /barriers. The grey inner ring show core stages in a strategic planning policy cycle. The outer rings highlight the specific landscape (green) and mainstreaming (purple) opportunities. The outer black line links these opportunities together to show that whereas these opportunities maybe more important at specific stages in the strategic planning process, they are important and relevant throughout the strategic planning process. The inner “red cloud” shows cross-cutting barriers to strategic planning for nature. Annotations to the right of the figure expand on the landscape and mainstreaming opportunities/barriers identified

The workshops have illuminated and reinforced the strategic planning tensions identified earlier in the paper associated with short termism (Van Dijk 2021), uncertainty (Hillier 2016) and changing power relations (Allmendinger and Haughton 2010). Perhaps most significant in the context of this paper there was a new but important tension exposed relating to the universal rejection of a separate strategic planning process that champions nature in favour of more holistic approaches incorporating and managing economic, social and environmental interests from the outset which endorses the pursuit of mainstreaming and landscape-scale approaches. It was also noteworthy that both workshops were operating within the shallower model of mainstreaming moving away from positions demanding more radical change, challenging existing power structures (Trygg and Wenander 2022). This reinforces Currys (1993) fallacy of creeping incrementalism by adding layers of governance to current arrangements rather than rejecting current policy and governance frameworks as not fit for purpose. However, as Scott et al., (2022) recognise more radical change pathways may initially start within more shallower models before progression to more radical pathways ensue. Here other drivers of change may cumulatively operate and impact. Trying to understand these driver impacts and the role that mainstreaming and landscape-scale concepts and associated mechanisms might contribute is illuminated in Fig. 5 as a conceptual representation of the workshop outputs set within wider mainstreaming and landscape-scale challenges and opportunities. At the centre are the key cross cutting tensions and barriers emerging from both workshops and the literature which impact on different stages in the policy cycle (from vision to evaluate). For the most part, these provide barriers which need to be overcome. Within this linear policy cycle at particular stages, we have then identified both joint and bespoke mainstreaming and/or landscape-scale opportunities and challenges. This diagram is now unpacked and justified with reference to our workshop results and wider literature.

### Common ingredients needed for realising mainstreaming and landscape-scale opportunities

Both landscape-scale and mainstreaming concepts embed more holistic and system thinking perspectives including multifunctionality, interdisciplinary job roles and placemaking/placekeeping remits that challenge existing silo mentalities (Fig. 5). In the absence of any single agency with an integrated remit tackling economic, environmental and social agendas collectively at a strategic level, partnerships have evolved as one of the principal delivery mechanisms for mainstreaming and landscape-scale goals. However, to be successful they need to be designed and maintained as inclusive and cross sector partnerships for the long term to tackle strategic problems holistically. Currently, too many nature initiatives are addressed within siloed agencies or environmental partnerships with key interests such as planning health and regeneration excluded (UKNEAFO 2014). Partnerships need to involve and engage with wider environmental economic and social interests from the outset, set within improved understanding of respective positions investing in conflict management and knowledge exchange strategies and tools (Cowling et al 2008; Scott 2012). Here effective leadership and communication are prerequisites being able to mobilise diverse audiences and crucially identifying a common vocabulary around terms that unite such as placemaking and placekeeping (Fish et al. 2015; Scott et al. 2018). Too often sectors will champion their own conceptual vocabulary rather than translate them in order to build bridges with others. Furthermore, there is a trend to simply add new partnerships creating additional layers of governance complexity to navigate (Scott 2012: Workshop 2). Whilst these may generate initial interest, they rarely last given time and resource demands. Thus, investing in improving and diversifying existing partnerships becomes a better strategy but one where leadership becomes crucial in being able to mobilise support across different audiences as well making sure the partnership generates actions and long-term commitments (Kitchen 2000) as highlighted in the Greater Manchester Combined Authority model.

Effective leadership is a key opportunity to drive forward mainstreaming and landscape-scale agendas and implement change if delivered within a trustworthy, legitimate, transparent and mandated governance framework (Lockwood et al. 2010; Senbel 2015; Hersperger et al. 2019). In 4 English case studies, Scott et al. (2018) found that nature was better mainstreamed where there was strong leadership evident at both political and chief officer level working together to shape the culture of the organisation. In Greater Manchester Combined Authority, a pioneering form of landscape scale devolved city-regional governance championed the natural environment through green infrastructure and wider natural capital investment (Haughton, 2020; Workshop 2). Here leadership had multiple dimensions, via a democratically elected mayor with visible leadership of portfolios such as strategic planning and by local council leaders empowered as deputy mayors. Additionally, its leadership has gained trust through the development of multiple public–private partnerships (Harding 2020). In England at least, these governance models may be important opportunities to enable strategic planning, which can bypass some of the barriers national politics presents, and thus also operate on a scale to enable the landscape scale and enable mainstreaming opportunities.

Our workshop results mirror the literature with a concern over disintegrated governance with, seemingly, no one body with sufficient authority or scope to operate at this level, thus inhibiting leadership except within emerging combined authorities as part of a complex devolution process where relatively few areas have devolved planning powers (Scott et al. 2013).

The need for a strong mandate for either voluntary or statutory approaches is important given the recognised neglect for the natural environment in planning decisions, compared with economic interests (Workshop 1: Scott et al. 2018). Voluntary approaches with innovative vision and broad participation can still suffer from issues of legitimacy (Mäntysalo et al. 2015). For example, the Sacramento Area Council of Governments in California developed a consensual voluntary regional plan but failed on local delivery (Allred and Chakraborty 2015). In the Nordics, it has been argued that the success of informal strategic planning relies on the balance between accountability, inclusiveness, liberty and fairness, set within an independent framework to avoid a parallel system (Mäntysalo et al. 2015).

Conversely, statutory strategic planning has a strong mandate through legislation as recognised in both workshops, helping to level the playing field (Workshop 1). However, control without democratic input can erode trust. This has been attributed to the abolishment of regional governance in some European systems (Tait and Hansen 2013). Legitimacy of such statutory strategic planning can also be weakened when there is a lack of transparency and therefore accountability (Falleth et al. 2010). Therefore, strategic planning incentives need to be seen as participatory, democratic and accountable from the outset in governance arrangement (Gains 2015: Workshop 1).

There was also a tension about planning for uncertainty and the lack of tools available (Schon, 1971). There was concern from both workshops that the existing spatial planning tools were not being used to their full potential a situation compounded by the plethora of new tools that were being added to by academics. In particular, the impact assessments and CIL/Section 106 tools needed to be used earlier in the policy process to deliver multifunctional gains for policy or developments rather than being seen as hurdles to overcome to secure plan approval (Workshop 1). Additionally, they needed to be redesigned to address cross boundary issues, issues of cumulative impact as well as enabling stronger community input (Bice (2020).

### Mainstreaming challenges and opportunities for strategic planning processes and outcomes

The multi-scalar politics of planning plays a fundamental part in shaping the opportunities and challenges for mainstreaming nature and the nature of change. Trygg and Wenander’s (2022) Swedish study found that planners lacked awareness of political agendas or the tools or capabilities to prioritise policy trade-offs. This resonates with workshop results set within a consistent relegation of nature with vague and uncertain policy objectives (Allmendinger and Haughton 2009). This reflects a post-political neoliberalist turn with a shift away from direct government intervention and service provision towards more market-based interventions, driven by the imperative to deregulate, liberalise trade and investment, marketise, and privatise natural resources, ultimately leading to fragmented governance (Olesen 2014). Here, the concepts of ecosystem services, natural capital and nature-based solutions have now become established in policy but attract significant concern from those who challenge the anthropogenic assumptions built into them urging a shift towards more radical conceptualisations of nature with people as opposed to for people (Spash and Hache 2022; Mercado et al. 2024).

One of the key tensions currently inhibiting strategic planning as shown in Fig. 5 is the nature and impact of power relationships. This is a complex and perverse tension in England and elsewhere, compounded in particular by multi-scalar conflicts. For example, where national government usurp local authority control through the creation of additional governance layers (Fearn and Davoudi 2022). In the 1980s, the Thatcher administration created development agencies which were given increasing powers and budgets, stripped away from local authorities. In 2010, the Conservative- Liberal Democrat coalition abolished strategic planning at the regional level stigmatising discourses around its interventionist and anti-growth characteristics and dismantling all associated structures, which were deemed to be New Labour clothing (Bafarasat and Baker 2022). In its place, local enterprise partnerships were created commanding significant resources away from local authorities. The Oxford Cambridgeshire Arc provides a recent example of a government-led development scheme that due to political headwinds catalysed public opposition eventually diluting central government commitment to priorities elsewhere (Valler et al. 2023). Such political ebb and flow suggest strategic planning is highly vulnerable to changing political ideology as echoed by Workshop 1 and 2 participants who wanted the political dimension more exposed.

Furthermore, and notably since the workshops, the incremental changes of BNG and local nature recovery strategies in England from the Environment Act 2021 confirm the pursuit of neoliberal thinking within a shallow mainstreaming approach rather than any fundamental realigning of natures’ values in policy changes. Indeed, recent government announcements have reigned further back on green agendas due to perceived issues of cost and equity.

### Improving strategic planning outcomes for nature at the landscape scale: Generating multifunctionality and identifying trade-offs

The landscape-scale champions multifunctionality at a range of spatial scales all working in harmony with each other from neighbourhood to local to regional (catchment) to national within a range of different plans. All too often those scales do not work together as illuminated by the political tensions discussed earlier. A lot of landscape-scale work has revolved around the use and evaluation of ecosystem services, set within the natural capital approach. Yet all too often the necessary trade-offs that are required in policies and plans are not identified or legitimised in a meaningful and transparent way, alienating particular stakeholder interests particularly where landowner options become constrained by landscape-scale designations (Hamback et al. 2023). The summary principles of the Lawton report “More, Bigger, better and joined up" provide a template for landscape-scale work but size and connectivity seem to be the principal concepts from wider literature reviews (Donaldson et al 2017) and are reflected in the emergence of local nature recovery strategies in England.

## Conclusions

This paper was built on the need to improve strategic planning for nature but rather has exposed an inherent and fundamental contradiction requiring its reframing as to how to embed nature, economy and societal issues collectively in strategic planning processes from the outset in both policy and practice. This finding brings into play both mainstreaming and landscape-scale concepts as they offer more holistic thinking and tools that help us make progress in strategic planning outside the current silos that fragment and disintegrate planning. Set within a wider desire of strategic planning more generally in both process and outcome terms, there is an urgent need to rediscover and embed the core ingredients of strategic planning moving away from the disintegrated governance we currently experience due in no small part to neoliberal traditions and protection of the status quo. This paper has identified some mechanisms to achieve this; effective leadership; building bridges through a more inclusive vocabulary supported by more inclusive partnerships. Currently strategic planning is inhibited by political tensions and our workshops have focused on the shallower mainstreaming pathways rather than arguing for more radical or transformative pathways. However, this does not signal failure but rather a fluctuating journey where we need exemplars and models that champion greater collaboration and cooperation moving away from the competitive short-term conflict between departments and agencies which so often stifles strategic planning.

### Supplementary Information

Below is the link to the electronic supplementary material.

Supplementary file1 (PDF 785 KB)

## Footnotes

1 No names of people are disclosed here given ethical protocols. Rather the workshop is named for ethical and confidentiality reasons.

2 Permitted development rights allow applicants to perform certain types of work without needing to apply for planning permission. They derive from a general planning permission granted not by the local authority but by Government.

3 The Planning Inspectorate are charged with assessing the soundness of local plans and to that end several recent decisions have ruled against plans for lack of alternative sections.
