# Supplementary material for: Improving strategic planning for nature: Panacea or pandora’s box for the built and natural environment?
Source: Ambio. 2024 Mar 15;53(8):1136–51. doi: 10.1007/s13280-024-01995-9 (PMC11183010; doi:10.1007/s13280-024-01995-9)
Supplement: Supplementary file 1 — Supplementary file1 (PDF 785 KB) [file 13280_2024_1995_MOESM1_ESM.pdf]

Ambio

Electronic Supplementary Evidence A, B and C

Title: **Improving strategic planning for nature: panacea or pandoras box for the built and natural environment?**

Authors: **Alister Scott and Matthew Kirby**

Supplementary Evidence A

Briefing Note for ADEPT Strategic Planning workshop: Welcome to RUFshire

Introduction

Good strategic planning is necessary to address key societal challenges we face such as climate change breakdown, declining biodiversity and declining health and well-being, together with the need for more, affordable and better housing and securing economic growth. However, whilst we may have the theory and tools to enable this, the delivery is often weak. All too often we tend to identify, diagnose and treat strategic planning challenges separately within established sectoral and professional silos which can all too readily lead to perverse outcomes and disintegrated development. Thus we need to identify the key ingredients that make good strategic planning.

From an academic review the following core ingredients feature prominently.

- **Change management:** Crucial to managing change is the co-development of a shared vision with strong leadership. Here change should be managed to best advantage reflecting the public interest.
- **Co-design and coproduction:** This is usually pursued within a partnership model thus diverting from conventional top down approaches to engage across multiple public(s) (ideally not just the usual suspects), but often managed within existing power relations with only incremental change.
- **Integration across different sectors and scales from different agencies**: This is pursued within a set of wider spatial and placemaking objectives. integration across different sectors and scales from different agencies. Key here is that horizontal (sectoral), vertical (spatial) and temporal scales are dealt with holistically and simultaneously.
- **Evidence-led proportionality:** Here there is a focus on requiring proportional evidence and data which is assessed to help prioritise outcomes;
- **Effective Communication:** Within strategic planning key messages need to be adapted for effective communication to different audiences reflecting their specific needs as one size does not fit all;
- **Investment:** Strategic planning as a process should create outputs that lead to new opportunities to lever funding based on key hooks
- **Delivery:** Using bundles of *regulatory*, participatory and incentive tools to help deliver and manage change including long term stewardship.

These ingredients are important as they will then inform the workshop exercise. As senior managers involved in place-based approaches and placemaking I want to capture your experience and expertise through taking you out of your current work environment to work within a new hypothetical space called RUFshire.

Your mission will be to rescue the rather ad-hoc approach to planning that has taken place in URF and RUF district councils over recent years. This has been exacerbated by the lack of progress made on a joint local plan (now abandoned) and effective placemaking. **Your table has been seconded by MHCLG to help shape an effective strategic planning process for the two councils with the development of a strategic plan as envisaged under the NPPF 2019 revised[1].**

Background to RUFshire

Whilst the following material is background information to the area, you are encouraged to use your collective imagination and improvisation based on your own experiences collectively as a table to bring RUFshire to life. This does give you freedom to translate your current challenges into RUFshire.

Tables will have a large detailed aerial photo of the area but an electronic version is here http://www.participology.com/images/hpic1.jpg

Please view the 28 questions that give you a feel for the pressures in the area. https://www.dropbox.com/s/lpf5wj7hvtrih4d/ADEPT.docx?e=1&dl=0

This area has suffered significantly in austerity with many similarities to the situation facing NE England. RUFshire has suffered a loss of its historical car manufacturing base and has been trying to reinvent itself for many years but without any significant external investment (as of yet). Development has tended to focus on derelict land and the quarrying legacy which is scattered throughout the area. New development has not been helped by the green belt which is seen as strangling the future expansion of the southern Gateway of RUFshire. Currently land prices are very low and the area has significant deprivation and derelict industrial estates.

More recently in 1999 St Ruf was established as a garden village (within green belt). This has attracted new executives to the area who then commute outside the area to work. However, with superfast broadband, there are a large number of people able to work from home with the nearby AONB providing a high quality and accessible natural environment.

The rural areas have been struggling. There is a declining farming base due to pressures from farming in the peri urban spaces with the villages acting as magnets for those who aspire to a rural life but with easy access to a city. Flooding is a real problem from the AONB which has been badly managed and overgrazed.

In terms of the housing market it is estimated that some 30,000 new dwellings are required over the next plan period. Rural villages have strongly resisted any new housing and the green belt poses a challenge to meet need. The declining industrial base has given rise to a spate of green technology ideas and plans but with no specific proposals or investors. This area specifically around Rufhampton is not deemed attractive due to its high crime rate and deprivation.

There is also real concern over the decline in biodiversity. The Special Area of Conservation not in favourable condition (great crested newts) and whilst small pockets of wilder nature are present in abandoned quarry workings ,the quality of the natural environment generally is of concern. The canal is also suffering from pollution and neglect in places although ambitious plans are being made (Canals and Rivers Trust) to promote a recreational trail.

[1] https://www.gov.uk/guidance/national-planning-policy-framework/3-plan-making pars 20/21

Workshop Questions and Actions.

Prior to the meeting it would be helpful if you could have done the following drawing from your own experiences.

1. Critically review the core ingredients of strategic planning listed on page 1
2. What BIG idea do you have to improve strategic planning outcomes in your own area (please bring this on a post it note) to the meeting.
3. Read the different scenarios of pressures and opportunities in RUFshire to understand challenges and opportunities.

The workshop will be structured around small group tables enabling you to collectively identify and record:

1. What are the core ingredients of a good strategic planning process?
2. What are the key strategic policy challenges for RUFshire emerging from the information?
    a. How might these inform the new joint strategic plan
    b. How might these inform a duty to cooperate (statement of common ground) between the two councils[2]?
    c. What are the key nature conservation challenges.
3. How might the challenges for nature conservation be addressed with respect to policy and delivery on the ground?
    a. What key tools might be used
    b. Who needs to be involved.

[2] The statement by South Downs national park might be of interest as well as drawing upon your own experience. https://www.southdowns.gov.uk/wp-content/uploads/2018/04/SDLP11-Duty-to-Cooperate-Statement.pdf

Supplementary Evidence B
What Does Good Strategic Planning for Nature Conservaiton Look Like?

Introduction

This is a summary of discussions at a joint ADEPT workshop using RUFshire as a hypothetical strategic planning prompt on 27th January 2020.

The results are presented in a bullet form narrative with some comments, links and questions as appropriate

****

Questions

The following 4 questions formed the basis of the workshop and the responses from flip charts have been bulleted with a supporting narrative based on discussion.

1 What are the core ingredients of a good strategic planning process?

- **Political**
- **Positive change management**
- **Co-design and coproduction: (participatory)**
- **Integration across different sectors and scales from different agencies**
- **Evidence-led proportionality:**
- **Effective communication**
- **Investment:**
- **Delivery**
- **Leadership**

These were all seen as valuable components but as **tools.** They need to be seen within the national, regional and local **POLITICAL contexts** that act as strategic umbrella and drive their use/misuse. Understanding the governance frameworks and scope for subsidiarity were crucial cross cutting ingredients.

Need to focus on the **OUTCOMES** and **GOALS of** what you are trying to achieve and the challenges being addressed. We see all too often separate plans and strategies. BUT: How do they fit together; for example the 25YEP, NPPF and SDGs have issues of their integration. In addition there is the wider problem of disintegrated development with nature as a bolt on.

Regulatory tools were seen to be very helpful for nature conservation. But there is a danger that these only become a minimum standard that needs to be reached. Biodiversity Net Gain becomes

a new ingredient for strategic planning as it is becoming mandatory. Opportunities also exist within Duty to Cooperate and Community Infrastructure levy but currently nature is way down the list of priorities and danger with duty to cooperate is that it has become somewhat toxic.

However, it is also important to address **strategic issues through ambition as well as through vision**. Here the value of strategic planning forums formed due to need not legislation reflecting that there are many built environment professionals who value and champion strategic planning. However, key to issues of successful strategic planning is working across different scales simultaneously with strong leadership.

2. What are the key experiences and lessons for strategic planning for RUFshire?

The bullet points highlight the umbrella issue of disintegrated development. Housing numbers are perceived to dominate the placemaking agenda from national priorities which distorts other actions. The importance of securing buy in with elected members and other stakeholders raises the importance of engagement and building partnerships and codeveloping plans, policies and interventions. Working with unusual suspects was also important to develop innovative solutions. This all helps wider coordination and delivery where communication was seen to be fundamental. In building partnerships a more positive and consensual approach to planning was desired. Here the framing of nature as an asset was important. We also need to secure baselines and to monitor our interventions more effectively.

The key lesson is that nature is an integral part of decision making and needs to be inputted early into decisions using established and trusted evidence. Here the resource and skill base needs to be strengthened.

- Don't start the conversation with housing numbers. Need to understand the bigger picture of connections and interdependencies first.
- Need to know what are the joint priorities and outcomes why are you doing this at the outset and invest in this process with stakeholders and publics.
- Secure political buy in from outset and work across political divides given short term nature of administrations. Political obfuscation is a big problem.
- Need to work across silos and improve understandings of different sectors. Nature conservation can also be a very effective silo
- Be aware what you are doing and what others are doing -improve communication within and across different agencies. (often within can be the more difficult)
- Role of building effective and long term partnerships ; need good leadership and champions who can unite,.
- Need stronger worded policies to ensure that nature conservation has clout in decisions.
- Focus more on who is doing the delivery and what and where are the resources; too many strategies developed but not delivered.
- Monitoring becomes important. To understand impact of interventions.
- Be prepared to things differently (develop odd new partnerships and coalitions and develop joint solutions.
- Work from a clear national vision. (not always present)
- Review of green belt toxic and anachronistic; need planning to be seen more positive and consensual.
- Major development sites need to include ALL infrastructure:

- Do we collect too much evidence that distracts? We need to use our evidence base more effectively for baseline and long-term monitoring.
- Duty to cooperate is seen to be a failure but could be a hook to try and work across political divides on issues like climate change as a hook.
- Strategic tools like CIL raise never enough to cover main priorities like education and highways. Nature loses out.
- Skills and resource deficit in local authorities to develop plans. Heavy reliance on consultants.
- All growth is not good growth
- Need more consistent methods to identify need across different sectors. Role here for environmental limits. Here natural capital baselines become important.
- Good to see biodiversity net gain becoming mandatory but how do you achieve it. Lack of guidance.

3. Outcomes:

Natural capital, green infrastructure are both seen as the collective glue to help deliver improved nature conservation outcomes. Key to realising this is the governance frameworks. Elected member and community structures (assemblies) were seen as vital steps to co develop and identify the outcomes that were desired. **The process by which the outcomes are identified, matters**.

The main outcome was centred on improving biodiversity but set within a wider connectivity discussion which included health and wellbeing, transport (walking and cycling). The summary tapes also indicated here that the outcome needed to be more joined up rather than have siloed nature based outcomes. **Here the links to economic and social gains were important reinforcing a more system based approach**.

- Integrating role of local plan as a hub through which other plans and strategies can emerge.
- Better use of CIL and 106 for strategic planning outcomes.
- Development of a green infrastructure strategy as nature conservation glue. Nature based solutions as the delivery mechanism,
- Importance of outcome of increased biodiversity but set within wider connectivity theme that covers access and landscape.
- Enhancing environmental performance through natural capital re-generation and opportunity plans. Natural capital plans help join up nature and economy. (eg. Ox Cams)

4. Interventions: How might the challenges for nature conservation be best addressed with respect to policy and delivery on the ground

This has been split based on flip chart content.

a. Governance interventions

The focus here was about improving the integration between different plans and policies with agencies working collectively across placemaking functions. The importance of contemporary policy hooks was recognised; here both climate and biodiversity emergencies offer political and public traction.

Within existing NPPF the duty to cooperate was seen as an opportunity and threat. Some views identified its toxicity with regard to housing numbers but others felt it could offer potential depending on what emerges from the environment bill building on the climate emergency. There was concern over the powers and resources of LEPS and LNPs with widely varying performances across the country.

- The duty to cooperate has a potential role and could be strengthened using the outcomes of the environment bill
- Holistic approach across all government agendas perhaps using climate change and biodiversity emergencies as hooks. UNSDGs really should have a stronger foothold in the NPPF. However also need a political champion who can unite.
- Avoid too many disparate plans being produced. Need a coordinating plan (local plans?)
- Joint members forum help address cross cutting issues
- Role of LEPS and LNPS: different spatial geographies and also different outcomes and resources. How can these produce better outcomes and are these fit for purpose.
- Local plans need to have stronger policies but critically dependent on the evidence base to back them up (resource limitations). Who funds?

b. Interventions on improving public engagement

Public and political engagement was seen as a vital delivery tool. But it often lacked upfront investment (funding deficit) with a focus on consultation. The adversarial nature of planning did not help and it was seen as important to have external facilitators to help in difficult and contested situations. Nature was seen as an early priority that could help drive more positive and consensual discussions towards a shared vision. However, these would be helped by regulations such as biodiversity net gain for example. Interestingly, neighbourhood plans were seen as useful mechanisms but needed to be made much simpler.

- Role of an external facilitator in helping drive a process within safe managed spaces. Important to invest on what the outcomes are and the key challenges before a plan is produced. Rarely enough resources invested in upfront engagement. Consultation dominates.
- Stakeholder forums with ability to influence and inform decisions and policy; danger of tick box syndrome.
- Potential role for nature conservation as a mechanism for initial community engagement; easier to get agreement on a vision or set of outcomes.
- Neighbourhood plans get 35% of CIL in some areas. Are neighbourhood plans a good positive mechanism to join up public views. Need to be a simpler process however.
- Need funding sources to help invest in behaviour change. Not funded from capital funds yet crucial to improved success with nature conservation

c. Interventions using strategic tools

Natural capital was seen as generally positive here although concern over the multitude of terms and range off different tools available. Natural capital accounts could be used to highlight the value of green assets and interventions when compared with more traditional grey interventions. Biodiversity net gain becoming mandatory offered another tool that had strategic potential but interestingly a lack of guidance for its successful operation was causing concern. CIL also had

potential but was limited through the funds it could raise. Spatial variation in levies capability was critical. The role of Sustainability Appraisal was also mentioned and it reflected the need to use existing tools rather than try an develop new ones. Here the challenge was to integrate natural capital into these rather than create yet another additional check for local authorities to do**.**

- Biodiversity net gain and community infrastructure levy might allow for more strategic sites to be identified. Wider issue of offsetting and mitigation
- Role of natural capital accounts to demonstrate the value of green assets but also initiatives and interventions under the umbrella of nature based solutions. Important to have baselines to measure and monitor. Need resources for this.
- Role of developing a tool for identifying environmental limits ; link into viability.
- Neighbourhood plans as positive tools but how to scale up strategically.

d. Interventions to improve investment in nature

The answers here revolved around building from the industrial strategy (circular economy) with natural capital accounting seen as a key driver. Additionally, a key area to work on was tax changes to affect local economies and incentivise brownfield land and to levy charges on green field sites. Preferential rates can also accrue from enterprise zones but it was recognised that such sites should have minimum standards for urban natural capital. Finance should be secured through innovative partnerships which could result in sponsorship under CSR from big companies keen to promote their green credentials but also could lead to more innovative payments for ecosystem service schemes . Notwithstanding this the preparation of GI strategies was still see as key to highlighting how nature based solutions could be delivered in simple language.

- Corporate sponsorship from industry
- Promote social value and community benefits from nature: target messages to different audiences.
- Circular economy models.
- Change tax structures to benefit local economies. Levy on green belt development used to fund brownfield land. Tax emptions on brownfield sites.
- More Enterprise zones introduced but these need to have minimum requirements for urban natural capital
- Mixed public private payments for ecosystem service schemes.

e.. Interventions to improve success

Here there was a desire to use demonstration case studies to highlight success and learn lessons. This was seen as key to motivate key stakeholders. Reports rarely do. Learning lessons from mistakes was also highlighted as important but often difficult to secure.

- Best practice review of why did that plan work/didn't work well for persuasion of communities, politicians and other stakeholders.
- Demonstration examples but need to be living labs
- Look at wide examples such as Copenhagen building 7k timber homes on derelict land
- Best practice review of how to deliver successful biodiversity net gain and wider environmental gains.

.

Supplementary Evidence C
What Does Good Delivery of Strategic Planning Look Like forNature?
Special Invitation Workshop: Report of Discussions

Introduction

This report is a summary of a workshop held in University of xxxxxx London on 28th January 2020. Five tables were created; each with a specific challenge. The catalyst for this workshop was the joint publication of "Understanding our growing environmental vocabulary in England: Connecting Green Infrastructure, Natural Capital, Ecosystem Services and Net Gain(s)within the English Planning System.

Table 1: How consideration of environmental net gains could complement biodiversity net gain planning? Francis Randerson Defra
Table 2: How can a natural capital approach improve spatial planning processes and outcomes when working at the landscape scale? Sarah Trouw Environment Agency
Table 3: How do we best integrate and deliver evidence-led nature based solutions into existing plans and schemes for the long term? Juliet Staples Liverpool City Council
Table 4: How to get the environment properly valued in business case appraisal using regional business case templates? Andy Brown University of Leeds
Table 5: How can strategic plans/policies as envisaged under the NPPF improve outcomes for the natural environment? xxxxxx University of xxxxxxxxx.

The report below summarises the main discussion points and outcomes. As well as the discussions the big ideas/challenges have been highlighted and where possible embedded into the separate table reports. These have also been developed into a set of recommendations. Throughout the material I have added reference to useful links with some questions posed as appropriate.

ACTION: It would be really useful if you could read through the set of materials and add your own thoughts; links to examples and also develop/amend any recommendations. As I said in the workshop this will enable a further iteration.

Preliminary recommendations

1. The workshop revolved around the **need to think more holistically about how the different terms associated with nature conservation such as natural capital, ecosystem services, green infrastructure, nature recovery networks and net gain relate to each other in both**

theory and practice. Separate table feedback highlighted the need for exemplars and examples of good practice. However, it is recommended to use best practice examples that bring together all these terms within research, policy and practice interventions rather than have separate exemplars. Investigate how far both South Downs, Liverpool and Greater Manchester can feature in this; together with the examples provided by Oxford Cambridge Arc and Liverpool Urban Green up in the workshop. Actions for follow up within narratives to improve the conceptual framework presented.

2. Defra have managed a series of different pioneer projects that have looked at different experiences with regard to nature conservation policy practices with a strong emphasis on natural capital and ecosystem services. It is important that we can fully digest the lessons emerging from these as soon as possible. A light touch evaluation is available here, it will be important to learn lessons from the conclusions of these to better inform this work. Actions for Defra Pioneer project lead.

3. There was strong support for regulation to strengthen nature conservation interests and the example of mandatory legislation for Biodiversity Net Gain was welcome. However, there was also a recognised need for improved national guidance. In particular, opportunity spaces were identified for strengthening the duty to cooperate to include nature conservation priorities. It is recommended that NPPG is updated to include how the duty to cooperate should necessarily include natural capital and key components of the environment Bill duties. Actions: ALL To identify if any other LPAs have DTC statements that cover environmental aspects like the South Downs National Park: Defra/MHCLG consider scope for improved guidance on statements of common ground.

4. It is recommended to examine the scope for guidance that can highlight how natural capital-ecosystem services-green infrastructure- nature recovery networks -nature based solutions- net gains can fit together using model policies for strategic plans in order to ensure that nature conservation is better joined up.

5. To provide a useful library of resources to help with the themes from the table discussions from both research and practice. I have made a small stab at this with links throughout this document.

6. There were calls for tools to be streamlined and to focus on embedding latest environmental thinking into existing planning tools. We identified duty to cooperate and tables did mentions Community Infrastructure Levy, Tax Incremental Financing and Sustainability Appraisal (any others?). I think it may be useful to collate examples of good practice here.

7. The business case for nature is not yet mainstreamed in planning investment decisions. It is important to capture how best to do this. The ICASP project is currently researching this through case studies and an assessment of capability to change combined authority business case templates to mainstream green and blue infrastructure.

8. Monitoring was a key issue across tables. It may be useful for recommendation 1 to have a monitoring component. All too often examples of good practice are "domesday" captures

at a moment in time. Thus it may be useful to use a living laboratory approach where specific interventions such as south Downs DTC model are monitored in terms of the

Generic Issues arising from the table discussions

- The need for better and more joined up thinking and governance. Danger of incremental change producing yet more complexity and disintegrated environmental policy hindering mainstreaming.
- The need for regulation for nature conservation to put it on an equal footing with other sectoral interests like biodiversity net gain (welcome) but needs guidance and support to achieve it.
- The need to support shared visons and objectives across multiple publics with regard to the type of natural environment needed.
- The need for better engagement with public(s) and building of partnerships across political, policy divides
- The need for better monitoring of policy interventions
- The need for simpler messages and communication of the science to policies and practice.
- The need for demonstration policies and practices within case study examples that can highlight what "good" or success actually looks like.
- The need for communicating better metrics and targets in terms of actions at multiple scales
- The need for better communication of environmental outcomes.

Generic Ideas from participants (post it notes)

- We have moved from a paradigm of protection to one of recovery with seemingly strong support. Challenge is how to deliver amidst multiple terms , tools and approaches.
- How can we enable a systemic change that sees nature conservation and nature based solutions embedded and implemented in all relevant policy
- More certainty and stronger wording in policy and enforcement; so the expectations are clear.
- Limit the growth of frameworks and tools and converge on a recommendation approach for appraising NC/GBI projects.
- A single nationwide GIS platform to show the spatial extent of all environmental plans and strategies
- Missing piece of environment bill governance – local environment improvement plan – integrating all parts of the environment in a way that treats environment as a system and provides a way for people to engage.
- Resolving economic growth GDP vs local well being dilemma not sitting in your natural capital/ES/GI /NBS silos. Are we nearly there yet or lost. Ie will any of this generate real change on the ground or jut plans and strategies that will gather dust on the shelves.
- Need publicly available and accessible demonstrators : can we have case studies showing clearly how the different concepts are applied together in one place.

- Ensuring that green infrastructure and nature conservation is a key objective in joint strategic spatial plans.
- Need joined up thinking and the best of market practices
- Lots of different approaches NCA, EA, ESA Need to integrate these. Introduction of Local Nature Recovery Solutions the way forward? ? If so landscape led methodology to achieve nature recovery could be utilised across the country – can be applied not just in protected landscapes. Use Glasgow approach
- Bring the right stakeholders together to enable local target setting and delivery within a nationally consistent framework

Table 1: How consideration of environmental net gains could complement biodiversity net gain planning

Relevant Big Questions

- **How can you manage and intervene sensibly in terms of net gain if it will and does cut across mitigation hierarchy.**

- **Lots of different approaches Net Gain, NCA, EA, ESA Need to integrate these; otherwise creeping incrementalism. Is introduction of Local Nature Recovery Solutions the way forward? ? If so landscape led methodology to achieve nature recovery could be utilised across the country – can be applied not just in protected landscapes. Use Glasgow approach**

- **Reskill and upskill all planners /members and all engaged in planning system on importance of and understanding of metrics relating to environment net gain and outputs**

Summary of Table Discussions

1. **ENG is a framework for development, not an end in itself.**
   a. **ENG policy should cover both natural capital assets as well as ecosystem services**.
   b. The relationship between Biodiversity Net Gain (BNG) and ENG was an area of contention.
      i. Some participants felt that BNG should be treated as a subset of ENG as ENG considers delivery of biodiversity outcomes as part of a longer list of ecosystem services that could be delivered. However **the majority view of other participants was that systems which are based on biodiversity (fully functional ecosystems) will deliver all ecosystem services most effectively and sustainably**, whilst treating biodiversity as a subset of wider ENG will be less resilient and sustainable and therefore deliver ecosystem services less effectively, with the collapse of the system being more likely.
      ii. Some participants felt that BNG delivery could conflict with wider ENG (examples such as poor water quality resulting in greater populations of wintering waders on the coast, which could be seen as trading off water quality outcomes for biodiversity outcomes). However other participants felt that prioritising the restoration of the structure and functions of the ecosystem would support the sustainable restoration of biodiversity and the ability of natural capital assets to provide other ecosystem services.
      iii. **BNG doesn't appear to work from a marine policy point of view,** it isn't applicable to marine planning. ENG is more applicable.
      iv. Issue of understanding local context where simply trying to achieve greatest biodiversity gains might conflict with environmental justice considerations.
   c. **ENG shouldn't just be applied to new development**, protection and enhancement of the natural capital assets and ecosystem services in existing developments is important too.

d. Local environment strategies may be necessary to show what needs to be protected and enhanced and opportunities for delivery of ENG contributions. LNRS may be useful here.
e. Well evidenced design guidance would help to ensure good quality ENG interventions.
f. **Role of planning system to provide some prioritisation of ENG dependent on evidence base and local context**. No one size fits all approach. Key is to have a transparent way to identify ES trade offs.

2. **It's a good idea, but the evidence is not always there (or adequate) to fully support it.**
   a. We cannot wait for all the evidence we might want to be available, we need to put the policy(ies) into place, and measure the delivery of it, allowing for the policy to be updated as needed when new evidence emerges.
      i. Iterative plans and policies would be ideal, to allow for 'adaptive management', but this may not be possible in many scenarios.
      ii. Implementation combined with monitoring will help us to understand what is optimal (although an element of the precautionary principle needs to be applied).
      iii. We are not always sure what aspects of biodiversity provide ecosystem services.
      iv. When evidence is patchy, this can risk bias towards what we do know or subjectively feel is important. This will need to be guarded against.
      v. Practitioners as well as academics should contribute to evidence gathering.
      vi. National Ecosystem Assessment is due in 2 years, this may be helpful.
   b. Monitoring delivery and the functionality of the nature based solutions implemented will be very important to ensuring that ENG is achieved; and that the interventions continue to be effective into the future.
      i. **Once a policy is in place, we should measure delivery retrospectively**, e.g. Marine Licensing post-consent monitoring.
      ii. Investment into local planning authorities and government departments to carry out and analyse the results of monitoring will be essential.
      iii. The results of monitoring need to be used to update and refine policy, as well as individual developments.
      iv. Effective, up to date and frequently updated habitat mapping is essential to support the monitoring of delivery and for Ecometric. NRW's habitat mapping may be a good example to investigate.
      v. Technological innovations are not the full answer to monitoring though, it needs to be recognised that effective monitoring requires personnel on the ground.
      vi. Policy could require developers to make contributions to a monitoring fund to support the monitoring needed to effectively implement the policy.
      vii. NERC could be encouraged to fund more monitoring (currently it's not considered a funding priority for them).
   c. ENG is for the long term, nature based solutions and adaptations often take time to establish so that gains can be seen and monetary benefits realised, so there will be a challenge to demonstrate success for short term government who are pushing for short term economic gains, but it's important we find a way to do this.

3. **Financial input will be needed to develop and implement ENG policy.**
   a. Treasury's support is needed to ensure that the finances required to implement the policy are available.

i. A business case for Treasury should be produced.
ii. We need to be able to demonstrate the benefits of the policy to private investment.
iii. For more private sector money to come into the system there needs to be a spatial framework which everybody can relate to on the same basis.

b. How can we create opportunities and incentives for natural capital assets, in a way which supports the right assets in the right places?
    i. Consolidation and connections – we need a better understanding of where money flows and opportunities to bring private money in.
    ii. Ideas - sell carbon credits, BNG credits? 'Stack' the benefits (to discuss with Treasury on Monday)?
    iii. Is there a role for ELM?
    iv. Private offsets?
    v. Work with developers to come up with good designs for ENG, incentivise the best designs, financially or by other means?

4. **Effective governance and join up within Government departments will be important to success.**
    a. Short government terms encourage short term thinking, but an ENG policy requires us to look into the long term and plan for natural capital and ecosystem services which will work way into the future.
    b. It is positive that MHCLG and DEFRA are working together, further Cross-government co-operation will be essential.
        i. BEIS will need to be included.
        ii. The driver for planners is to enable sustainable growth. Marine planning delivers cross-departmental objectives but the priority for terrestrial planning is currently housing. When the government departments are not joining up, so there are different drivers, how do you lever the Treasury for them to value ENG?

    c. Policies will need to be carefully written and with stakeholders where possible to encourage maximum buy in, polycentric governance?
    d. ENG should be included in overarching governance policies to ensure that it is not confined to certain policy areas.
    e. Reporting will be important to ensure accountability
        i. An Environmental Net Gain register similar to the BNG register will be needed.
        ii. Office for Environmental Protection could have a role.
    f. MMO have a policy on ecosystem services already, join up with MMO will be important to ensuring that an overarching policy works in the marine environment and to learn from their experience in developing policy in this area.

5. **Delivery of ENGs for all natural capital assets and ecosystem services will usually not be possible, so effective prioritisation is important.**
    a. ENGs that tackle the climate and biodiversity emergencies and have human health and wellbeing benefits should be prioritised for delivery (multifunctional green infrastructure).
    b. We must remember that we are working with dynamic systems, so we can't be too prescriptive.

c. Prioritisation decisions should consider the whole system, not individual natural capital assets and ecosystem services, or we risk delivering for certain natural capital assets or ecosystem services whilst impacting others without a proper understanding of the implications.
d. Prioritisation should also link to what people want.
    i. This can be established through methods such as the Ecosystem Approach.
    ii. The Ecosystem Approach has been trialled already so the results of this would be helpful.
    iii. South Downs National Park plan could provide a best practise example.
    iv. Local prioritisation should be informed by regional, national and international priorities as well as those which are specific to the local area.
    v. We should aim to better capture and promote the economic and social effects of environmental gains to inform local stakeholders and galvanise support.
e. Prioritisation should reflect the opportunities offered by the local environment.
f. Is sound and noise landscape included in this? E.g. Changes in sound-pressure impact on air, therefore is that linked to ecosystem services?

6. **Reskilling of a range of different groups will be needed to implement an ENG policy.**
    a. Development of capability to measure natural capital assets and ecosystem services across relevant sectors.
        i. Includes both the BNG and any ENG metrics.
    b. Development of the capability of planners and other relevant professionals to explain to the public the relevance of the ENG elements of any given development to them.
        i. Development management are output focused. Re-skilling council members of the importance of ENG is particularly important to making and communicating development management decisions.

Table 2: How can a natural capital approach improve spatial planning processes and outcomes when working at the landscape scale?

Relevant Big Questions

- Lots of different approaches NCA, EA, ESA Need to integrate these. Introduction of Local Nature Recovery Solutions the way forward? ? If so landscape led methodology to achieve nature recovery could be utilised across the country – can be applied not just in protected landscapes. Use Glasgow approach
- Limit the growth of frameworks and tools and converge on a recommendation approach for appraising NC/GBI projects
- A single nationwide GIS platform to show the spatial extent of all environmental plans and strategies
- Effective protection for existing assets. \Make their value visible . Link into multifunctional nature recovery networks that define both biodiversity and ecosystem services.

Summary of discussion

Natural Capital Planning is as much a Process as an outcome; an underpinning /overarching approach rather than a one size fits all approach.

To utilise NCA properly for decision making you need to have clear objectives and you need to support people properly in setting these objectives. It is Important to realise there are multiple users of this approach and we need to recognise this in the tools we develop and provide.   It is also important that you have the buy in from senior politicians and decision makers early in the process so they can in effect champion it.

So for example a local authority in a local plan context ; the key is how to align this NCA so it is not seen as something extra to do; it works with existing processes and systems.

Duty to cooperate ; NCA does it translate to this obligation or are their rules of policy or interventions to facilitate that to make the process work. The example discussed on Table 5 of the South Downs highlights how the outputs from a NCA or ecosystem services assessment can lead to the identification of strategic priorities

Need for outputs and decisions/interventions made to be transparent and so be able to work backwards from that and to have confidence in it. Raises wider issues of governance and legitimacy which are key to pursuing NCA as evidenced from Defra marine pioneer.

The importance of the evidence base behind any NCA process and the issues and resources needed for the refreshing and updating of the data and how robust the data was. Often dealing with out of date data.

Talked about language and the need for common levers for change. In the natural environment there are lots of different terms being used creating difficulties for different audiences. Key issue around audiences and how you talk and communicate with different audiences. A one size fits all approach rarely works. Need to tailor outputs to different audiences explicitly.

Overlaying the language and toolkits around the capacities and capabilities of different audiences

Overcoming the Silos mentality: there was a danger of the evidence in every natural capital plan being siloed and only used by environmental voices; question of how to embed that within business interests . There was a wider issue of mainstreaming.

How does NC mapping allow you to scenario plan and understand trade-offs to inform better decision making.

Taking a natural capital approach about long term change and investment. So how can we incentivise people/business to engage with it when it seems so far removed.

Recognise the need for a formal land use planning system to make this work ; ie not compartmentalising activity. Need to join up land use with development in the planning system

Nature recovery networks can create tensions between different stakeholders for example between rewilding and more managed solutions.

In any area of new activity you need best practice examples and pilots. It thus becomes very important to draw out the lessons from the defra pioneers who were looking at the NCA particularly through the urban pioneer in Manchester. If NCA is going to work we need good examples of best practice to show how it works and to show the additionality and net gains it brings,

Be more spatially explicit; what's in it for me ; be as simple as possible in explaining how it can be used and with what benefits and what do challenges does it solve.

Table 3: How do we best integrate and deliver evidence-led nature based solutions into existing plans and schemes for the long term?

Relevant Big Questions

- How can we enable a systemic change that sees nature conservation and nature based solutions embedded and implemented in all relevant policy
- How can we make the use of NBS more intelligence led. . can we use existing data layers (GIUS) to identify and prioritise local issues and use this to determine the most beneficial types of NBS to be protected or introduced for maximum benefits,
- Resolving economic growth gdp vs local well being dilemma not sitting in your natural capital/ES/GI /NBS silos. Are we nearly there yet or lost. Ie will any of this generate real change on the ground or jut plans and strategies that will gather dust on the shelves.

Summary of Discussions

What are the evidence sources available?

- GIS mapping as being used in Liverpool City Council Climate Just mapping which overlies environmental factors with social issues – Rowntree foundation
- University of Greenwich TURAS project with Stuart Connop was mentioned
- Unalab have an NBS catalogue to help determine selection of the most appropriate NBS
- Swansea GI strategy has some appendices which illustrate available data for areas quite well
- A single repository of tools for climate change assessment and ecosystem services would be helpful Ecosystem Knowledge Network
- NATURVATION EU project has just released a manual on Nature Based Solutions

How do we monitor NBS to demonstrate benefits?

- Urgently need right level of resources for long term monitoring (cross reference from table 5)
- It's important to collect the right data when monitoring
- Usually data collection is short term e.g. in Liverpool for 2 years but benefits increase as NBS matures;

- University of Portsmouth (John Williams) on the pro-suds project looks at site management over longer time scales
- The URBAN GreenUP project used the EKLIPSE KPI framework as an EU recommendation but a set of agreed and standardised UK climate change indicators or KPIs would be welcomed for comparison and benchmarking

How do we integrate NBS?

- Integrate into the work of others such as utilities, highways, parks, stakeholders etc.
- Co-benefits are important and so is the need to quantify the invisible multiple benefits. A common unit of £ value arguably is best to illustrate multiple benefits.
- Use Natural capital accounting to show where the financial value is for NBS interventions. Combine NCA and NBS to get the best investment for outcomes.
- It is also possible to lever money for NBS by asking what is in the current engineering maintenance plan that would be saved or reduced by NBS thus allowing that to be freed up to invest into NBS.
- Issues over how we integrate at both strategic and project (local level). Strategic documents at combined authority level may supersede some of the NBS detail we have worked hard to integrate locally unless this is checked and addressed (important cross reference to table 4)

What do we need to make it easier to mainstream delivery of NBS?

- A simple route map and guidance of how to do this for Local Authorities, developers and stakeholders
- Training for local authority staff in other areas such as highways. Drainage, planning, regeneration etc
- A means to share lessons learnt
- The ability to make a robust economic case during a period of local government funding cuts and staff losses

Table 4 How to get the environment properly valued in business case appraisal using regional business case templates

Relevant Big Questions

- Need joined up thinking and the best of market practices.
- Limit the growth of frameworks and tools and converge on a recommendation approach for appraising NC/GBI projects.

Summary of Discussion:

Appraisal Process

- Currently dominated by transport economics

Evidence and its Robustness

- Concerns that even if you gain buy-in from stakeholders for a policy, programme or project with significant Green and Blue Infrastructure (GBI) benefits, it falls down during the appraisal of the economic case because your evidence for GBI benefits is not robust
- May be a potential tension between national priorities and local priorities (e.g. flood risk) – so perhaps an explicit approach is needed such as a geo spatial evidence base
    - Is there scope to review how different ecoservices benefit different areas?
    - Concerns that an accepted value in one area would not be applicable for all areas of the country

      How to value green and blue infrastructure and biodiversity?

- Can we operationalize Natural Capital in the 'strategic case' of HMTs '5 case model' for Business Cases, especially for large (non-marginal) impacts of GBI?
- Can we develop the 'system of systems' approach in the 'strategic case' to aid GBI inclusion?
- Argument that it is also possible to perform a cost-benefit analysis on a system of systems in the 'economic case' of HMTs 5 case model: e.g. as happened in transport sector where previously heavy emphasis on travel time and now much more complex system is recognised
    - Therefore if ecosystem services were measurable we could put more emphasis on the value of the environment
- Discussion surrounding the difficulty in calculating the benefits of GBI
- Discussion that people are concerned about the cost of GBI as there is not a huge amount of evidence and can be hard to see and quantify the benefits of GBI
    - Could a framework be produced to help understand benefits and costs of GBI?
    - Can tools surrounding Natural Capital Evaluation be used to create more certainty around the benefits and costs of GBI?
- Value extraction vs value creation – what are stakeholder interests; how do you generate buy-in from stakeholders (such as developers); how does this fit with the business case?
- Possibility that buy-in could be brought about by greater certainty in the planning permission – or from increases in house/land value due to GBI?

Large Programme/Project vs Small Project/Intervention

- Is a strong strategic narrative required to produce a Business Case at the level of every intervention (or small project) or is strategic case more important for large (regional and national) programmes?
- If LAs and smaller bodies often don't have capacity to produce extensive Business Cases – is there a middle ground to be determine where there is guidance for completing the strategic case and for the economic case with the ability to provide more indicative figures?

Table 5 How can strategic plans/policies as envisaged under the NPPF improve outcomes for the natural environment?

Relevant Big Questions

- Incorporating robust standards into green infrastructure strategies and local plan policies.
- Ensuring that green infrastructure and nature conservation is a key objective in joint strategic spatial lands.
- How can we enable a systemic change that sees nature conservation and nature based solutions embedded and implemented in all relevant policy
- More certainty in policy and enforcement ; so the expectations are clear.
- Reskill and upskill all planners /members and all engaged in planning system on importance of and understanding of metrics relating to environment net gain and outputs
- Link ELMs into strategic planning.
- Strategic planning for functionality connected habitat networks at regional level

Summary of discussions

<u>Themes: Strategic, Visionary, consistency and Monitoring</u>

1. Need to be clear what we are trying to achieve in order to measure success. Monitoring is a key part of this - trying to identify small gains that are less frightening would possibly make it more achievable, and also in some instances more palatable.
2. Environment Bill priority areas creates an opportunity to reset the Duty to Co-operate in the NPPF so that a more strategic approach for the environment is drawn up, not just focused on housing. The approach used by South Downs highlights the potential. Agreed that regulation was a necessary focus to level up the playing field for the environment .
3. There is a need to join up all the separate silos within planning that are treated separately. Here infrastructure planning, land use planning, rural land management (ELMS) and utilities. The system needs to be more visionary, not reactive, and include a broader range of inputs - in particular utility provision. There is no point creating a natural habitat if it is dug up for an oil pipe or broadband cables.
4. There is a wider need to join up across and within local authorities who produce separate plans and strategies. It creates a messy environment. Is there scope for a strategic or local development plan to become the hub within which the spokes become other plans for the area.
5. Consistency in application of the policy is important, in particular what a sound plan looks like - a number of examples spoken about where PINS had arrived at different decisions on some of the newer priorities such as wellbeing. There needs to be a clear benchmark so good practice can be shared.
6. Viability should be considered at the outset at the plan level, not at the application level. Where viability cannot be achieved, this should trigger pump-priming of funding rather than driving down the local provision as this usually impacts negatively on environmental factors which are seen as costs.
7. Spoke about the benefits of ELMS - this will be increasingly important in delivering for both nature and people - ensuring that available land is used strategically.

Rough notes:

<u>Delivery</u>

Ensuring that there is consistency in the application of the NPPF across inspectors - as difficult to share best practice without a consistent message.

- Inspector examination approach - lack of consistency in applying the NPPF in relation to health and wellbeing
- Demonstrating in policies and plans how this applies to every individual (South Downs plan)
- Environment Bill targets - long term strategic targets (how do these filter down to the local level as they are national targets). What is the 'glue'? Duty to Co-operate - how is this filtered in? At regional level - strategic level - how are these used for meaningful outputs? Local plans need to be consistent. Currently in Hertfordshire revising GI strategy (9 years old). Messiness is hindering aligning the objectives
- Noise - how is this covered in the Environment Bill? Impacts differently on the environment than humans. Note Welsh Government action plan on noise and soundscapes at present.

<u>Plan-making and strategic planning</u>

A strong case for strategic plan at a national level but political appetite not there at present. Needs to be visionary, not reactive (as it is at the moment).

National planning needs to have all the players sat together - e.g. DCMS cable laying.

Local plans currently need to jump through the MHCLG/PINS hoop but Marine planning jumps through all hoops - terrestrial planning needs to jump through the same hoops.

- Distribution of housing needs to be addressed at the strategic level to enable the benefits to be realised. How does LNRS feed in to housing planning when it is at a local level?
- OxCam - housing in a water stressed area liable to flooding - but not considered when deciding where to locate housing, and water services not joined up (Ofwat not involved etc).
- No national spatial strategy - only country without one. Very difficult to strategically plan without energy, water etc. constraints known. Seen as politically dangerous to draw lines on maps. Issue of cost vs political pragmatism vs devolution
- Trees seen as having a moment - but other forms of habitat also very important. Consider where carbon sinks are located so air quality is improved where it is needed, not where there is cheap land. Cost of tree planting is high. Trees arms race will have huge cost and maintenance implications. Need to consider also impacts and trade offs for ES on where they are planted and for what priority.

- HMT modelling how to use public and private investment combined to deliver multiple benefits. What is the credit mechanism?
- Overarching policy at the top of the plan needed. Has to be pulled together. Embedding natural environment in the Duty to Co-operate.
- Need integration of systems - nitrates in Solent caused by agriculture but being dealt with by planning.
- Habitats issue - driven by regulation rather than aspiration.
- Need a clear view of how you want to benefit society to deliver an aspiration. What links them? A view of gain. Need to have both components
- **What does a sound plan look like?** Integrate all the sectors - environment, wellbeing, housing
- Inconsistency among inspectors - especially in new areas. PPG - ensuring clear messaging. Sharing good practice – ensuring

Utility point / <u>common objective -</u> currently out of step. Utility providers need to align their plans.

- Utility regulation is all about consumer pricing, doesn't align with planning policy/framework objectives.
- Need a common set of objectives for all infrastructure.
- Steve Q - NPPF is the map (but isn't a spatial map, which most people want to see)
- Without the map, we don't know where the problems are (e.g. HS2 will improve an already OK service, there are bigger issues such as the East Coast main line).
- Communities under huge amounts of pressure will not buy into ENG as they have bigger issues (e.g. buying food).
- Need to see the problem of climate change as an opportunity - more permitted growth system - incentivise BNG (e.g. fund business in locations that deliver good BNG).

Environmental Land Management Strategies (ELMS) a key element in making gains while meeting needs for development

- More of a narrative - what is the vision?
- Needs to show multiple benefits
- Ensuring OGDs don't draw a map without input from ecosystem services
- Needs to be broader than nature - ecosystem services.
- Want to think more boldly but missing the scale of the problem - centuries of damage but we aren't going to achieve this all at once. Small steps rather than big targets. Monitorable - e.g. 10 houses in one village rather than looking at 40k houses across the region.
- Natural England - do some monitoring but it isn't sufficiently resourced.
- MMO - relies on other sources of data.
- OEP - but would need funding to deliver

- Need to be broad about what the environmental gains are - e.g. opening up listed buildings
- Increasing automation of data
- Farmers - not happy about their diversification applications being rejected but no data available on what applications were refused and why
- MMO - SSSI data - needs upfront resourcing for monitoring
- Need HMT to understand that improvement is iterative, not linear. Need funding to pilot, demonstrate success and then roll out if successful or change the process/policy.

Duty to Co-operate:

How do we amend and use this as a tool for directing conversations/negotiations following the environment bill? Early engagement that involves all your providers is key - to set the vision that is deliverable.

Viability at plan level would trigger pump-primping funding and investment rather than driving down the local requirements.

- Amending the wording and strengthened for the natural environment.
- What does 10% mean in a sound plan? Regulation process needs to back the process up.
- Gain is about what will work for people in that place?
- Focus on what are the outcomes? What are we trying to achieve in each place?
- 10% on a site may not be best for the environment overall! Beauty/recreation/noise/social justice a factor on these sites especially for housing.

People had to be flooded 3 times before they believed they lived in an area that is at risk of flood (research by university).

Risk is a cross-cutting theme - risk of environmental damage, risk of climate change - including in the NPPF? How feasible is this but it is well understood by developers and business. However South Downs policy might be good here.

Relationships - LEPs/two tier councils - how well they work together determines

How do we evaluate the outcomes of local plans?

- Comes back to what is trying to be achieved?
